# Design and synthesis of a bis-macrocyclic host and guests as building blocks for small molecular knots

**DOI:** 10.3762/bjoc.16.192

**Published:** 2020-09-18

**Authors:** Elizabeth A Margolis, Rebecca J Keyes, Stephen D Lockey, Edward E Fenlon

**Affiliations:** 1 Department of Chemistry, Franklin & Marshall College, PO Box 3003, Lancaster, PA 17601, USA

**Keywords:** alkynes, azides, host–guest, macrocycles, molecular knots

## Abstract

The thread–link–cut (TLC) approach has previously shown promise as a novel method to synthesize molecular knots. The modular second-generation approach to small trefoil knots described herein involves electrostatic interactions between an electron-rich bis-macrocyclic host compound and electron-deficient guests in the threading step. The bis-macrocyclic host was synthesized in eight steps and 6.6% overall yield. Ammonium and pyridinium guests were synthesized in 4–5 steps. The TLC knot-forming sequence was carried out and produced a product with the expected molecular weight, but, unfortunately, further characterization did not produce conclusive results regarding the topology of the product.

## Introduction

Macrocycles have played a central role in the development of molecular recognition, self-assembled molecular devices, and molecular topology [1–6]. For example, early work by Pedersen on crown ethers [1], Lehn on cryptands [2], and Cram on hemicarcerands [3] demonstrated that preorganized macrocycles have the ability to act as hosts for various guest cations and compounds. Their seminal work was recognized with the 1987 Nobel Prize in Chemistry. More recently, the 2016 Nobel Prize in Chemistry was won by Sauvage, Stoddart, and Feringa for their work on molecular machines [4]. Sauvage [5] and Stoddart [6] extensively used macrocycles in their ground-breaking work on catenanes, rotaxanes, knots, and other topologically novel compounds. Exciting advances in the field of molecular topology continue with novel trefoil knots have been prepared with an all-hydrocarbon example by the Itami group [7] and the synthesis of a single enantiomer by Leigh’s group [8]. Complexity has also been achieved with recent work showing that eight-crossing knots [9–11] and a nine-crossing composite knot can be synthesized [12].

**Figure 1:**
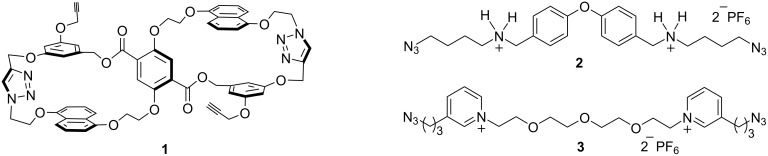
Structures of electron-rich bis-macrocyclic host **1**, and electron-poor guests bis(ammonium) **2**, and bis(pyridinium) **3**.

Herein the synthesis of a unique bis-macrocyclic host **1** is described (Figure 1). Host **1** was designed to be a second-generation building block in the thread–link–cut (TLC) approach to molecular knots [13]. The two 25-atom macrocycles are electron-rich and complementary to the electron-deficient guests bis(ammonium) **2** and bis(pyridinium) **3** (Figure 1). The electron-rich macrocycles of host **1** might also render it useful for other molecular recognition applications.

**Figure 2:**
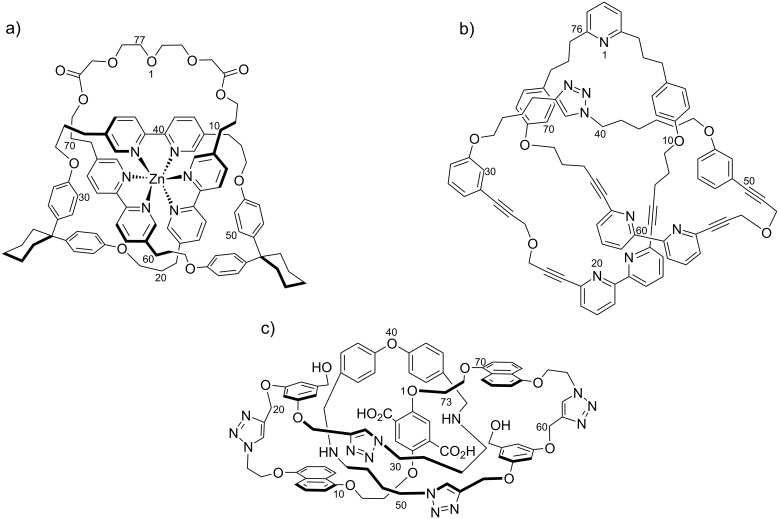
(a) Hunter’s 77 backbone-atom trefoil knot–metal complex [9]. (b) The world’s smallest knot: Leigh’s 76 backbone-atom trefoil [10]. (c) Target 73 backbone-atom trefoil knot of this work using host **1** and guest **2**.

A principle goal driving our second-generation TLC approach was to test the lower limit on the size of a molecular trefoil knot. In 2008 we surveyed the literature and suggested that a knot of 45–50 backbone atoms was theoretically possible [13]. At that time, the world’s smallest knot was an 80 backbone atom trefoil knot–metal complex by the Sauvage group [14]. Shortly thereafter, Hunter’s group published the synthesis of a knot–metal complex with 77 backbone atoms (Figure 2a) [15]. More recently, Leigh’s group disclosed the synthesis of the current record holder for the smallest knot, which has 76 backbone atoms (Figure 2b) [16]. Since the metal atom template has been removed, the Leigh compound is a true knot in strict topological terms [13,17–20].

The first-generation TLC approach for the synthesis of molecular knots involved a single knot precursor compound that had two macrocycles and two long tails [13,21]. Solvophobic effects [22–23] were used to promote the tail threading step, ring-closing olefin metathesis (RCM) was the linking step, and ester saponification was the cutting step. A potential problem with this approach was that the macrocycles were highly flexible and thus they might adopt a closed conformation hindering the necessary threading step. The flexible tails made the compounds soluble, but also appeared to hinder the formation of crystals suitable for X-ray diffraction to prove the topology of the product.

**Figure 3:**
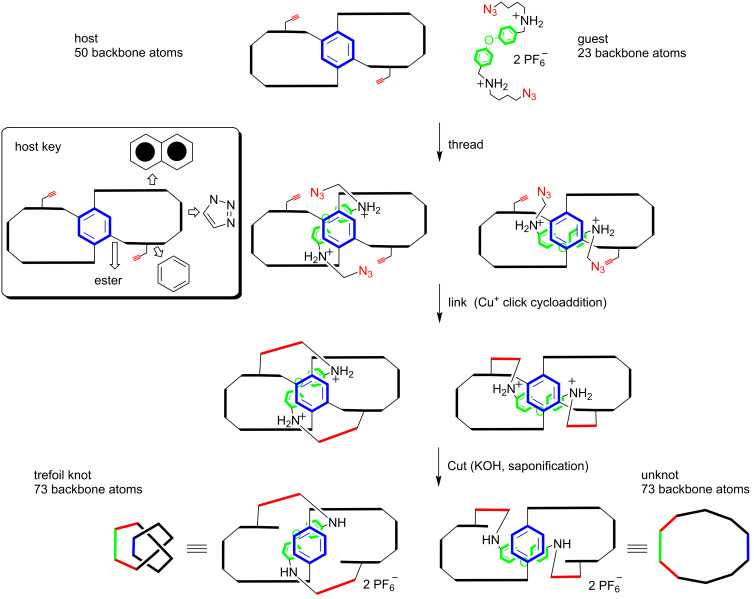
Schematic representation of the second-generation TLC approach to a 73 backbone atom trefoil knot.

The second-generation TLC approach to trefoil knots is presented here. It involves the binding of an electron-poor guest (**2** or **3**) in an electron-rich host (**1**) to promote the threading step, an alkyne–azide click cycloaddition as the linking step, and ester saponification as the cutting step [13,21] (Supporting Information File 1). The target trefoil knot using host **1** and guest **2** is shown in Figure 2c. The binding event during the double-threading step was modeled after previous literature precedents from the Stoddart [6,24–25], Gibson [26], Loeb [27], and Sanders [28] groups. Furthermore, host **1** was designed to be rigid so that the two macrocycles would maintain an open conformation which is required for the threading step. The approach is modular, such that one host can be paired with multiple guests in order to systematically explore the lower size limits of trefoil knots. For example, if TLC were successful with host **1** and guest **2**, then a 73 backbone-atom trefoil (and the corresponding unknotted macrocycle) would be formed (Figure 3 and Scheme S1 in Supporting Information File 1), whereas host **1** and guest **3** would lead to a 75 backbone-atom trefoil (and unknotted macrocycle).

## Results and Discussion

**Scheme 1:**
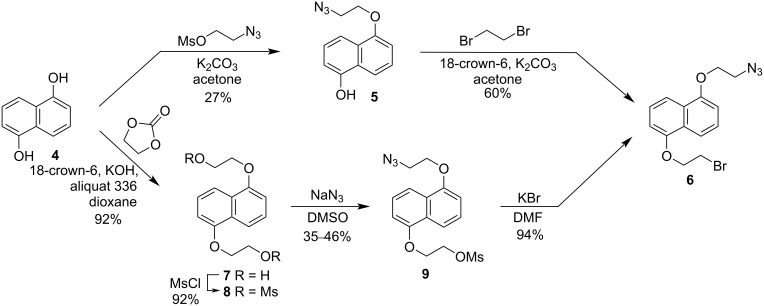
Two routes to azidobromide **6**.

The synthesis of bis-macrocyclic host **1** began by breaking the symmetry of naphthalene-1,5-diol (**4**) by alkylation of one of the alcohols with 2-azidoethyl mesylate to yield azide **5** in 27% yield (Scheme 1). Alkylation of **5** with 1,2-dibromoethane provided key intermediate azido-bromide **6** in 60% yield. This two-step route to **6** is efficient, but the 16% overall yield was lower than desired. An alternate route began by converting diol **4** to bis(2-hydroxyethoxy)naphthalene **7** in 92% yield by reaction with ethylene carbonate using a modified literature procedure (see Supporting Information File 1). Conversion of **7** to bismesylate **8** proceeded smoothly in 92% yield under standard conditions. The symmetry-breaking step in this route involved treatment of **8** with one equivalent of sodium azide in DMSO to give azide-mesylate **9** in 35–46% yield, which is reasonable based on a maximum statistical yield of 50%. Displacement of the mesylate with bromide provided a 94% yield of **6**. This route to **6** is twice as long as the alternative described above, but it is preferred because the 37% overall yield is more than twice as high and this route is more amenable to multigram scale reactions.

**Scheme 2:**
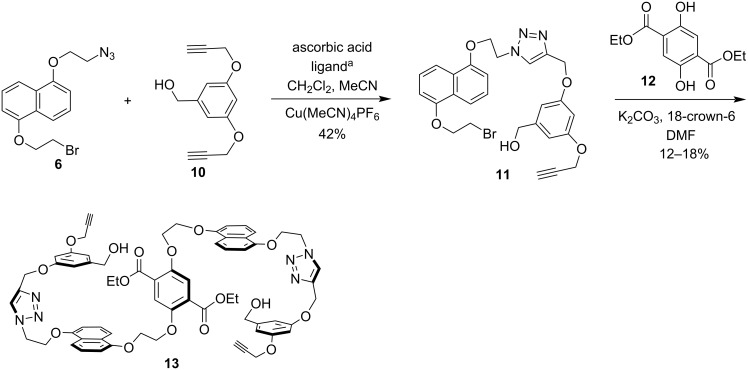
Initial route to core diester **13**. ^a^Ligand = tris(2-benzimidazolylmethyl)amine.

Azido-bromide **6** can undergo both alkyne–azide click cycloaddition and etherification and the effect of the reaction order on the overall yield was explored next. The click cycloaddition was pursued first and reaction of **6** with an excess of known (see Supporting Information File 1) dialkyne **10** under several of the most common conditions produced triazole **11** in only modest yields (Scheme 2). The best conditions involved using Cu(MeCN)_4_PF_6_ as the copper(I) source, tris(2-benzimidazolylmethyl)amine as a ligand, and ascorbic acid to provide a 42% yield of triazole **11**. Alkylation of diethyl 2,5-dihydroxyterephthalate (**12**) with **11** under standard conditions provided low yields (12–18%) of the core diester **13**, which contains all the atoms of host **1**. The yields for this route were disappointingly low, so it was hoped that changing the order of these steps would be beneficial.

**Scheme 3:**
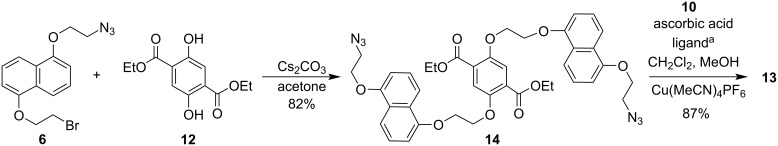
Better yielding route to core diester **13**. ^a^Ligand = tris(2-benzimidazolylmethyl)amine.

Alkylation of terephthalate **12** with azido-bromide **6** using cesium carbonate as the base provided high yields (82%) of core diazide **14** (Scheme 3). Click cycloaddition of **14** with an excess of dialkyne **10** also proceeded in high yields (87%) to give diester **13** which was identical to the material made by the route outlined in Scheme 2. The new route from **6** to **13** proved superior, as the yield for the two-steps improved dramatically from <8% to 71%.

**Scheme 4:**
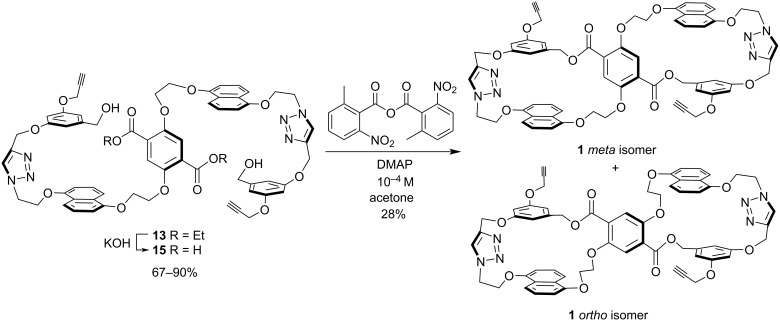
Saponification of **13** and bis-macrocyclization to form host **1**.

Saponification of diester **13** to diacid **15** was achieved in moderate-to-good yields (67–90%) and the ^1^H NMR spectrum showed the loss of the ethyl ester peaks (Scheme 4). Bis-macrocyclization of **15** under high-dilution using Shiina’s mixed-anhydride method [29] afforded host **1** in 28% yield. As with the analogous first-generation knot-precursor bis-macrocycle [15], host **1** was formed as a mixture of *meta*- and *ortho*-isomers. It is unknown which of the isomers is the major product, but the isomer ratio is approximately 2:1 based on NMR integrations of several aromatic peaks such as naphthlene signals at 6.7 (major)/6.8 ppm (minor) and the phenyl signals at 6.5 (major)/6.6 ppm (minor) and 6.4 (major)/6.3 ppm (minor). This is similar to the first-generation bis-macrocycle which had a 64:36 isomer ratio [15]. The macrocycles in host **1** are rigid by design (vide supra); however, this rigidity appears to also decrease their solubility. The ^1^H NMR spectrum was obtained in a dilute DMSO-*d*_6_ solution and, as expected, several of the aromatic peaks have shifted upfield relative to diacid **15**, because of shielding from nearby aromatic rings. The high-resolution mass spectrometric data and IR spectra also support the successful synthesis of **1**.

**Scheme 5:**
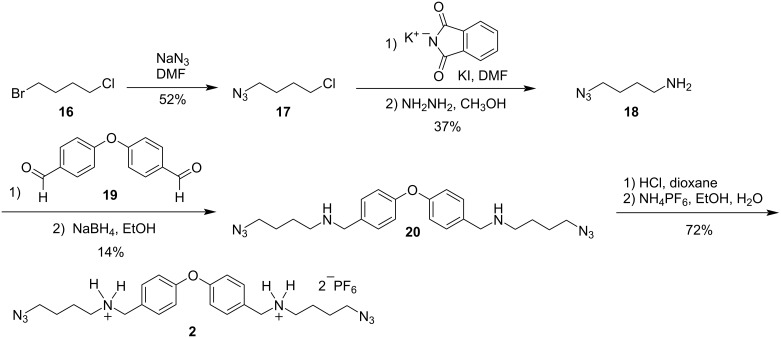
Synthesis of 23 backbone-atom bis(ammonium) guest **2**.

The synthesis of bis(ammonium) guest **2** began with selective displacement of the bromide in 1-bromo-4-chlorobutane (**16**) with sodium azide to give azide **17** in 52% yield (Scheme 5). Treatment of **17** with potassium phthalimide and catalytic potassium iodide was followed by hydrazine unmasking to give the desired aminoazide **18** in 37% over the two steps. Reductive amination of **18** with dial **19** provided diazide **20**, albeit in low yield (14%).

Protonation of **20** with HCl followed by anion exchange yielded 72% of the hexafluorophosphate salt of guest **2**. The ^1^H NMR spectrum supports the ammonium ions of **2** as all peaks shifted downfield relative to the chemical shifts in neutral diamine **20**. As expected, the methylene groups closest to the nitrogen atom shifted the most (>0.8 ppm for each). The IR spectrum also supports the structure of **2**, as strong peaks for both an azide asymmetric stretch (ν 2099 cm^−1^) and a PF_6_^−^ stretching vibration (ν 845 cm^−1^) were observed.

**Scheme 6:**
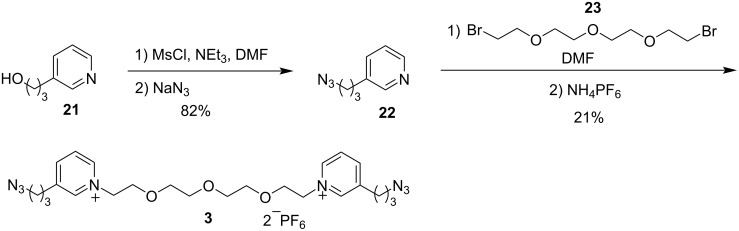
Synthesis of 25 backbone-atom bis(pyridinium) guest **3**.

The synthesis of bis(pyridinium) guest **3** started with the conversion of alcohol **21** into the corresponding mesylate followed by substitution with azide to give azidopyridine **22** in 82% yield (Scheme 6). Reaction of **22** with known [18] dibromide **23**, followed by anion exchange, yielded 21% of the hexafluorophosphate salt of guest **3**. The ^1^H NMR spectrum supports the pyridinium structure of **3** as the aromatic peaks shifted downfield by 0.6–1.4 ppm relative to the chemical shifts in pyridine **22**. The IR spectrum supports the structure of **3** as strong peaks for both an azide asymmetric stretch (ν 2102 cm^−1^) and a PF_6_^−^ stretching vibration (ν 835 cm^−1^) were observed.

To test the threading step, a ^1^H NMR experiment was conducted. A mixture of **1** and **2** was dissolved in a 1:1 acetone-*d*_6_/DMSO-*d*_6_ solution. The spectrum of this solution showed upfield shifts of 0.1 to 0.4 ppm for the methylene protons on the guest and smaller downfield shifts of 0.02 to 0.09 ppm for some aromatic resonances on the host. These shifts suggest at least some host–guest complex was formed, even in this competitive polar solvent mixture. The full TLC sequence was tested by reacting **1** and **2** (1:1 ratio) in a solvent mixture of acetonitrile/dichloromethane/methanol (1 mL/1 mL/0.1 mL) with [(CH_3_CN)_4_Cu]PF_6_ and ascorbic acid (see Scheme S2 in Supporting Information File 1). After removal of the solvent, the cutting step was performed by heating the crude product with KOH in a water/THF/ethanol mixture to saponify the esters. The pH of the mixture was adjusted to neutral by adding HCl and the resulting precipitate was collected. The IR spectrum of the crude reaction product no longer contained an azide peak, consistent with a successful click cycloaddition. The peaks in the ^1^H NMR spectrum in DMSO-*d*_6_ solution at room temperature (≈24 °C) were broad, whereas the spectrum at elevated temperature (e.g., 45, 90, 105 °C) had sharper peaks. This effect of temperature on the spectrum is consistent with restricted dynamics of the backbone such as reputation [30] or slow exchange between different conformers; however, both the trefoil knot and the macrocyclic unknot could exhibit this behavior. Two separate triazole peaks were observed (8.19 and 8.38 ppm), consistent with a new triazole ring being formed. The mass spectrum of the product showed peaks for the expected mass of the TLC product(s) and various cation adducts: (peak mass, assignment, relative intensity): 1563.9 Da, [M + H]^+^, 88; 1584.9 Da, [M + Na]^+^, 28; 1601.9 Da, [M + K]^+^, 52; 1626.8 Da, [M + Cu]^+^, 42. It is not surprising that the product would sequester metal cations given its electron-rich macrocyclic nature. Of course, the mass spectral data do not address whether the unknot, trefoil knot, or both were formed (see Scheme S1 in Supporting Information File 1). Repeated attempts to grow X-ray crystals of the product failed. To test for topological chirality, a europium chiral shift reagent was added, but the ^1^H NMR spectrum was too noisy to discern any peak doubling. Additional experiments, such as the TLC sequence with guest **3**, were not possible because of a lack of material. Future work will involve making a more soluble host so that NMR and chiral chromatography experiments can be conducted.

## Conclusion

The design of a second-generation thread–link–cut (TLC) approach to molecular knots was described. This differed from the first-generation TLC approach [15] in that the threading event was bimolecular, involving rigid electron-rich bis-macrocyclic host **1** and an electron-poor guest molecule (**2** or **3**), rather than unimolecular threading involving solvophobic forces. The flexible and modular approach involving several guests was designed to test the lower size limits on trefoil molecular knots, as the proposed TLC sequence can produce trefoil knots with 73 or 75 backbone atoms. Several synthetic routes to bis-macrocyclic host **1** were explored and the optimized route required eight steps and proceeded with a 6.6% overall yield. Bis(ammonium) guest **2** was synthesized in five steps with a 1.9% overall yield and bis(pyridinium) guest **3** was prepared in four steps and 17% overall yield. The TLC sequence using host **1** and guest **2** produced a product with the expected molecular weight, but the data were inconclusive on whether this was the unknot, trefoil knot, or a mixture of both.

## Supporting Information

File 1 Conformations of host **1**, TLC knot-forming scheme, experimental procedures and copies of ^1^H NMR spectra.
