# Supplementary material for: Design and synthesis of a bis-macrocyclic host and guests as building blocks for small molecular knots
Source: Beilstein J Org Chem. 2020 Sep 18;16:2314–21. doi: 10.3762/bjoc.16.192 (PMC7509378; doi:10.3762/bjoc.16.192)
Supplement: File 1 — Conformations of host 1, TLC knot-forming scheme, experimental procedures and copies of 1H NMR spectra. [file Beilstein_J_Org_Chem-16-2314-s001.pdf]

BEILSTEIN JOURNAL OF ORGANIC CHEMISTRY

Supporting Information

for

Design and synthesis of a bis-macrocyclic host and guests as building blocks for small molecular knots

Elizabeth A. Margolis, Rebecca J. Keyes, Stephen D. Lockey IV and Edward E. Fenlon

Beilstein J. Org. Chem. **2020,** *16,* 2314–2321. doi:10.3762/bjoc.16.192

Conformations of host 1, TLC knot-forming scheme, experimental procedures and copies of ^{1}H NMR spectra

License and Terms: This is a supporting information file under the terms of the Creative Commons Attribution License (https://creativecommons.org/licenses/by/4.0). Please note that the reuse, redistribution and reproduction in particular requires that the authors and source are credited.
The license is subject to the *Beilstein Journal of Organic Chemistry* terms and conditions: (https://www.beilstein-journals.org/bjoc)

Table of contents

- Anti and syn conformations of host **1**
- Proposed second-generation TLC knot-forming scheme
- Three different representations of the proposed trefoil knot
- Backbone numbering for small unknot (trefoil isomer would have the same backbone)
- Experimental procedures and compound characterization
- Copies of ^{1}H NMR spectra

Conformations of host 1

Host **1** has two rigid, electron-rich macrocycles. CPK models suggest that the macrocycles will remain in a relatively open conformation which should aid threading of the electron-poor guest molecules. The phenyl ring that has the propargyloxy substituent is free to rotate such that the two alkyne groups in **1** can adopt an anti or syn-conformation as shown in Figure S1 for the meta isomer of **1**. The same conformations are possible for the ortho isomer of **1** as well.

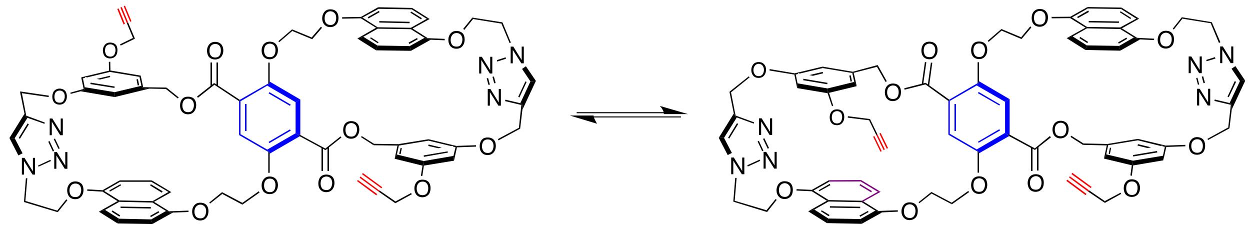

1 meta isomer
anti conformation

1 meta isomer
syn conformation

Figure S1: The anti and syn conformations of the meta isomer of host **1**.

Proposed second-generation TLC knot-forming scheme

Figure 3 in the manuscript shows a schematic representation of the thread-link-cut (TLC) sequence of electron-rich bis-macrocyclic host **1** and electron-poor guest **2** and Scheme S1 below shows a molecular representation of the sequence. The proposed threading step is promoted by electrostatic attraction of the ammonium groups with the electron-rich macrocycles as well as a potential hydrogen bond between the ammonium NH and a nitrogen or oxygen atom on the host. The linking step is a copper-catalyzed click cycloaddition reaction between the two alkynes on the host and the two azides on the guest. CPK models suggest the cycloaddition can occur on the alkyne of the threaded macrocycle (right side in Scheme S1) or on the alkyne of the other macrocycle (left side in Scheme S1). Finally, the cutting step involves cleavage of the ester linkage by saponification to release either the unknotted macrocycle (right) or the trefoil knot (left), depending upon which click reaction occurred in the linking step.

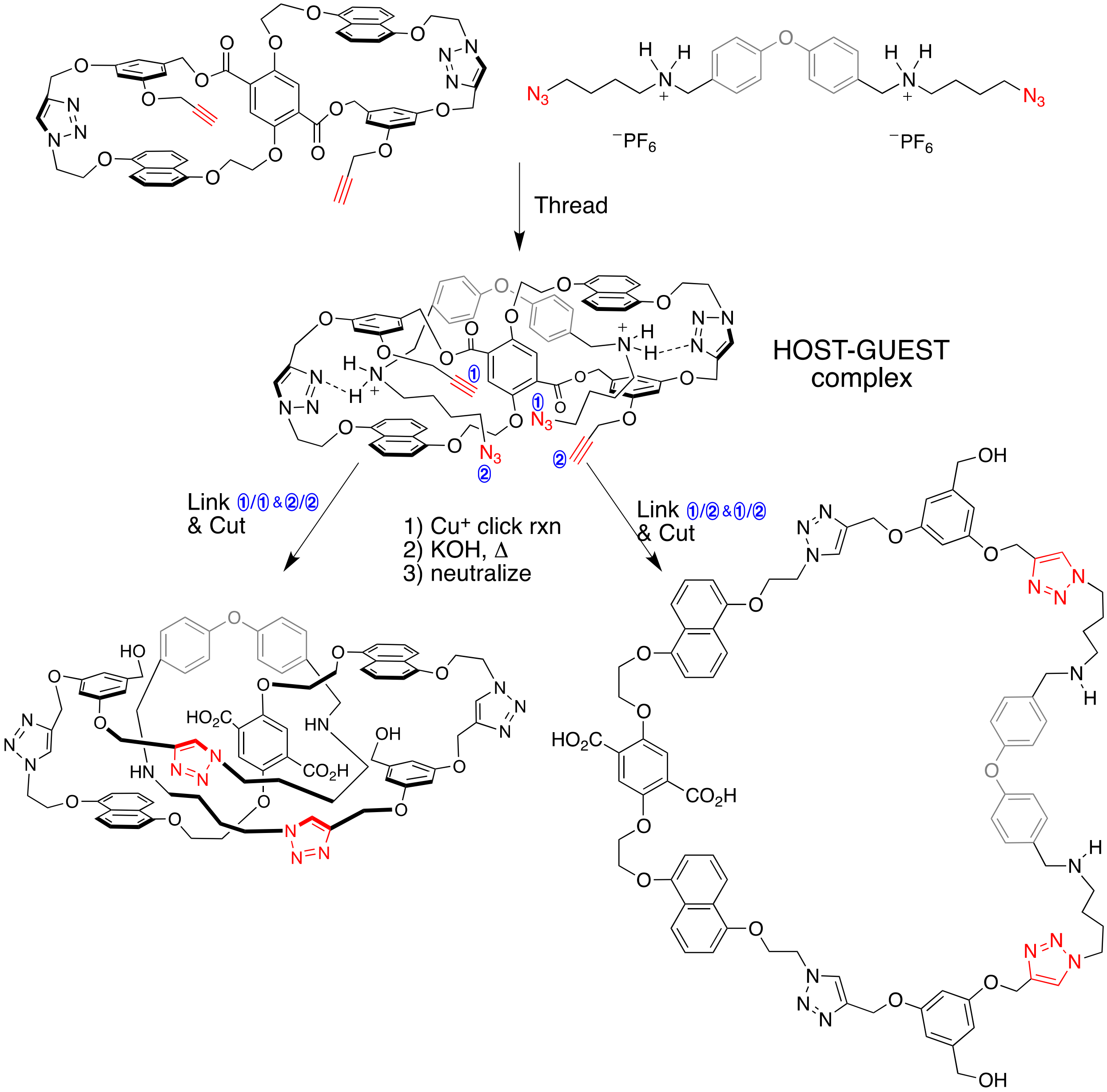

Scheme S1: Molecular representation of the second-generation TLC approach to small trefoil knots, see text for details. Click reactions between the alkyne **1** and azide **2** and alkyne **2** and azide **1** leads to the unknot macrocycle after cutting whereas click reactions between the alkyne **1** and azide **1** and alkyne **2** and azide **2** leads to the trefoil knot after cutting.

Figure S2 shows the proposed knot shown in three different ways to better illustrate the topology of the molecule. Figure S3 shows the backbone numbering for the unknot isomer that results when the TLC approach is successful for host **1** reacting with guest **2** (Figure S3a) or with

guest **3** (Figure S3b). The topologically isomeric trefoil knot would have the same backbone numbering.

a

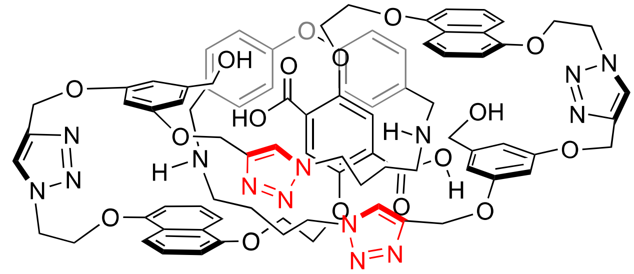

b

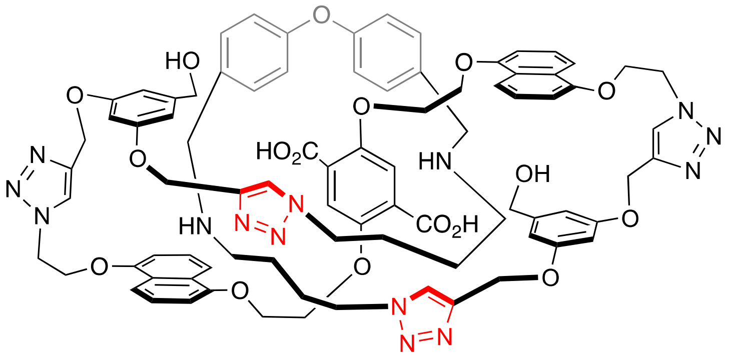

c

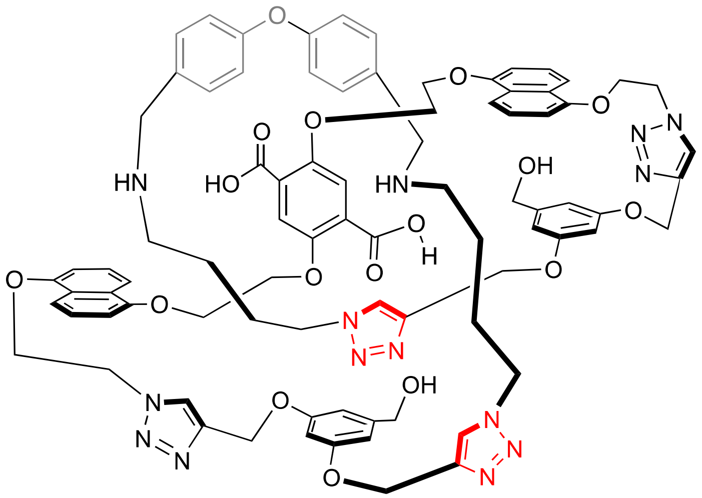

Figure S2: (a) Proposed knot with normal bond lengths. (b) Proposed knot with bond lengths exaggerated to make the structure more clear. (c) Proposed knot with bond lengths exaggerated and reduced to a minimum crossing diagram.

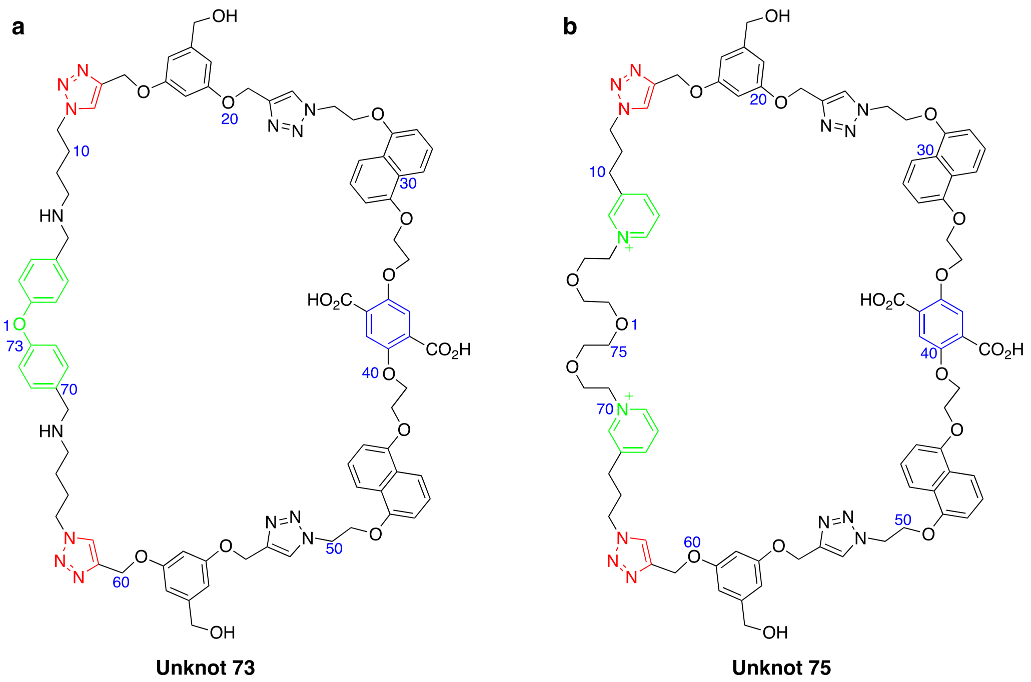

Figure S3: Backbone numbering for the unknots resulting from (a) the reaction of **1** and **2** or (b) **1** and **3**, see text for details.

Experimental

General

All reagents were ACS reagent quality, purchased from Aldrich, Alfa-Aesar, or TCI and used without further purification. 2-Azidoethyl methanesulfonate was synthesized by the literature method of Tookmanian et al. [1] 1,5-Bis-(2-hydroxyethoxy)naphthalene (**7**) was prepared by a modification of Dyer and Scott's[2] method, wherein potassium hydroxide, 18-crown-6, and aliquat 336 were used in place of benzyltrimethylammonium hydroxide. 3,5-Di(2-propynyloxy)benzyl alcohol (**10**) was prepared by the method of Antoni et al.[3] Azide **17** was prepared by the method of Yao et al.[4] 4-Azidobutylamine (**18**) was prepared by the method of Cunningham et al.[5] and matched the spectroscopic data reported by Carboni et al.[6] Dialdehyde **19** was prepared by the method of Yeager and Schissel.[7] Dibromide **23** was prepared by the method of Breyholz et al.[8]

All reaction mixtures were stirred with a magnetic stirring bar and conducted under a dry argon atmosphere unless done in an aqueous solvent system. Analytical thin layer chromatography (TLC) was performed on 0.2 mm silica plastic-coated sheets with F_{254} indicator. Preparative TLC was performed on 1 mm thick silica gel with F_{254} indicator. Flash chromatography was performed using 230–400 mesh silica gel. NMR spectra were obtained at the following frequencies: ^{1}H (500 MHz) and ^{13}C (125 MHz). Spectra were obtained in chloroform-*d* ($CDCl_3$) unless noted otherwise. Chemical shifts are reported in parts per million (ppm) and coupling constants are reported in hertz (Hz). ^{1}H NMR spectra in $CDCl_3$, DMSO-d_6, and acetone-d_6 were referenced to

tetramethylsilane (TMS = 0.0 ppm) as an internal standard. ^{13}C NMR spectra in $CDCl_3$ were referenced to the solvent peak at 77.0 ppm. ^{13}C NMR spectra in DMSOd_6 were referenced to the residual solvent peak at 39.52 ppm. ^{13}C NMR spectra in CD_3OD were referenced to the residual solvent peak at 49.00 ppm. IR spectra were obtained as a thin film (for solid samples) or neat (for oils and liquids) on an ATR instrument and the absorption peak frequencies are reported in cm^{-1} and intensities as very strong (vs), strong (s), medium (m), or weak (w). Mass spectrometry was performed at Franklin & Marshall College or at the University of Illinois School of Chemical Sciences, Urbana, IL and used electrospray ionization (ESI) in the positive ion mode unless noted otherwise. Mass spectral *m/z* peak data are reported in Daltons with a relative intensity to a base peak (base = 100). Melting points were measured on a Mel–Temp melting point apparatus and are uncorrected.

Abbrevations: ATR (attenuated total reflectance); brine (saturated aqueous sodium chloride); DMAP (4-*N,N*-dimethylaminopyridine), DMF (dimethylformamide); DMSO (dimethyl sulfoxide); EI (electron ionization); ESI (electrospray ionization); EtOAc (ethyl acetate); Et_2O (diethyl ether); FC (flash column chromatography using silica gel); MALDI (matrix-assisted laser desorption ionization); MeOH (methanol); PE (low boiling petroleum ether); TOF (time of flight); water (deionized water).

Procedures

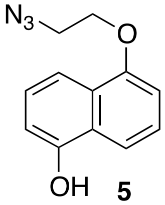
5

1-(2-Azidoethoxy)-5-hydroxynaphthalene (5). A mixture of 1,5-naphthalenediol (**4**, 5.00 g, 31.3 mmol) and acetone (100 mL) was degassed by argon bubbling for 8 min. Potassium carbonate (4.31 g, 31.2 mmol) was added and the mixture was stirred for 10 min. 2-Azidoethyl methanesulfonate (2.69 g, 16.3 mmol) was then added and the mixture was heated at reflux for 17 h. Additional 2-azidoethyl methanesulfonate (2.70 g, 16.3 mmol) was added and the heating was continued for 23.5 h. The solvent was removed under reduced pressure and hot 2.5% MeOH/CH_2Cl_2 (300 mL) was added to the residue. The salts were removed by vacuum filtration and silica gel was added to the filtrate, which was concentrated under reduced pressure. Purification on a large silica gel plug (0→60% CH_2Cl_2/PE) gave 1.915 g (27%) of **5** as pale yellow powder; mp 136-137 °C; IR ν 3274.4 (br, w), 2933.8 (w), 2878.0 (w), 2100.8 (m), 2086.2 (m), 2062.6 (m), 1600.3 (m), 1522.8 (w), 1414.3 (m), 1381.1 (m), 1283.0(m), 1269.4 (m), 1058.8 (m), 965.2 (m), 956.8 (m), 773.0 (vs); ^1H NMR ($CDCl_3$) δ 7.87 (d, *J* = 9, 1H), 7.79 (d, *J* = 9, 1H), 7.38 (m, 1 H), 7.32 (m, 1H), 6.85 (m, 2 H), 4.31 (t, *J* = 5, 2H), 3.72 (t, *J* = 5, 2H); ^{13}C NMR (~15% CD_3OD in $CDCl_3$) δ 153.73, 152.35, 126.65, 125.79, 125.54, 124.36, 114.95, 113.29, 108.88, 105.01, 67.04, 50.36; MS (ESI) 230.1 (M+1, 12), 202.1 (M+1-N_2, 100), 174.1 (M+1-N_3CH_2, 46), 172.1 (50); HRMS (ESI-TOF) calcd. for $C_{12}H_{12}N_3O_2$ $[M+1]^+$: 230.0930; found: 230.0931.

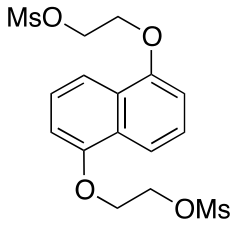

8

1,5-Bis[(2-methanesulfonoxy)ethoxy]naphthalene (8). A solution of **7** (8.83 g, 35.6 mmol) in DMF (90 mL) and triethylamine (15.0 mL) was cooled in an ice–water bath and methanesulfonyl chloride (7.1 mL, 91 mmol) was added dropwise over a 25 min period. The mixture was allowed to warm to room temperature and stirring was continued for 116 h. Methanol (4 mL) was added and stirring continued for 25 min. Then the mixture was poured into ice water (500 mL) and the tan precipitate was collected by vacuum filtration and washed with water. The pasty tan material was dried in an oven at 50 ˚C for 48 h to give 13.245 g (92%) of **8** as beige powder. mp 168-170 °C (dec.); IR ν 3034.5 (w), 2979.6 (w), 2946.1 (w), 1595.2 (w), 1514.2 (w), 1419.1 (w), 1401.5 (m), 1354.5 (s), 1330.1 (m), 1270.2 (w), 1181.4 (s), 1087.1 (m), 1075.9 (w), 1023.3 (m), 978.0 (m), 966.3 (m), 931.0 (s), 837.5 (w), 813.3 (m), 775.1 (s), 742.3 (w); ^{1}H NMR (DMSO-*d*$_6$) δ 7.79 (d, *J* = 8, 2H), 7.42 (t, *J* = 8, 2H), 7.03 (d, *J* = 8, 2H), 4.67 (br s, 4H), 4.42 (br s, 4H), 3.27 (s, 6H); ^{13}C NMR (DMSO-*d*$_6$) δ 153.32, 125.85, 125.59, 114.18, 106.22, 68.82, 66.16, 36.86; MS (ESI-TOF) 405.1 (M+1, 100), 382.4 (17); HRMS (ESI-TOF) calcd. for $C_{16}H_{21}O_8S_2$ $[M+1]^+$: 405.0678; found: 405.0667.

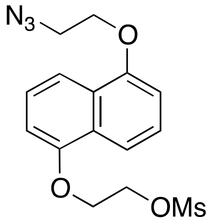

9

1-(2-Azidoethoxy)-5-(2-methanesulfonylethoxy)naphthalene (9). A heterogeneous mixture of **8** (10.0 g, 24.7 mmol) in DMSO (150 mL) was stirred and heated at 55 °C for 7 min until homogenous. Sodium azide (1.625 g, 24.99 mmol) was dissolved in water (4 mL) by sonication and then added to the homogenous DMSO solution. The mixture was heated at 60 °C and stirred for 18.5 h. The mixture was allowed to cool to room temperature. The product was extracted with water two times (150 mL), ethyl acetate two times (150 mL), and brine two times (150 mL). The combined organic layers were dried with Na_2SO_4 and concentrated to give 6.7814 g of a dark solid. The compound was dissolved in acetone/methanol/CH_2Cl_2 2:1:1 and preloaded onto silica gel. The column was eluted with 10% ethyl acetate/petroleum ether (1 L), 20% ethyl acetate/petroleum ether (500 mL), 30% ethyl acetate/petroleum ether (500 mL), 33% ethyl acetate/petroleum ether (1 L), and 45% ethyl acetate/petroleum ether (1.5 L). A beige powder was isolated and recrystallized from toluene to give 1.991 g (46%) of **9** as beige powder: mp 111.0-112.5 °C; IR ν 3061.3 (w), 3035.8 (w), 2982.8 (w), 2947.1 (w), 2877.1 (w), 2104.6 (s), 2071.0 (m), 1594.4 (m), 1513.4 (m), 1459.3 (w), 1416.7 (m), 1403.1 (m), 1388.3 (w), 1355.8 (s), 1330.0 (s), 1301.2 (w), 1270.3 (s), 1242.3 (m), 1183.5 (s), 1120.4 (w), 1086.0 (s), 1073.2 (m), 1022.2 (m), 977.8 (m), 965.7 (m), 931.9 (s), 839.3 (w), 819.3 (m), 811.2 (m), 771.8 (s), 746.5 (w), 632.4 (w);

^1H NMR ($CDCl_3$) δ 7.92 (d, *J* = 9, 1H), 7.88 (d, *J* = 9, 1H), 7.39 (m, 2H), 6.85 (d, *J* = 7, 2H), 4.71 (m, 2H), 4.42 (t, *J* = 3, 2H), 4.32 (t, *J* = 5, 2H), 3.73 (t, *J* = 5, 2H), 3.08 (s, 3H); ^{13}C NMR ($CDCl_3$) δ 153.86, 153.45, 126.58, 126.54, 125.35, 125.24, 115.31, 114.73, 1 05.94, 105.61, 67.88, 67.21, 66.11, 50.44, 37.84; MS (ESI-TOF) 374.1 ($M+Na^+$, 23), 352.1 (M+1, 35), 324.1 ($M+1-N_2$, 100), 228.1 ($M-C_2H_4OMS$, 52); HRMS (ESI-TOF) calcd. for $C_{15}H_{18}N_3O_5S$ $[M+1]^+$: 352.0967; found: 352.0950.

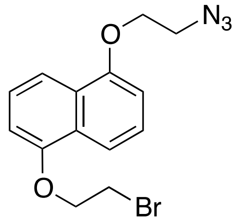

6

1-(2-Azidoethoxy)-5-(2-bromoethoxy)naphthalene (6).

Method A from 5: A mixture of **5** (1.92 g, 8.38 mmol), 1,2-dibromoethane (2.5 mL, 29 mmol), potassium carbonate (1.18 g, 8.55 mmol), 18-crown-6 (24 mg, 0.091 mmol), and acetone (30 mL) was heated at reflux for 18 h. Additional 1,2-dibromoethane (2.0 mL, 23 mmol), potassium carbonate (1.43 g, 10.4 mmol), and acetone (10 mL) were added and heating continued for 7 h. A final dose of 1,2-dibromoethane (2.0 mL, 23 mmol), potassium carbonate (1.00 g, 7.25 mmol), and acetone (10 mL) were added and heating continued for an additional 47 h. The mixture was concentrated under reduced pressure and the residue was partitioned between methylene chloride and water. The aqueous layer was extracted with methylene chloride and the combined organic layers were washed with brine, dried (Na_2SO_4), and concentrated under reduced pressure. The crude product was recrystallized from MeOH/toluene 4:1, followed by recrystallization from acetonitrile to give 1.695 g (60%) of **6** as yellow crystals; mp 107.5-108.0 °C; IR ν 3066.3 (w), 2937.7 (w), 2873.6 (w), 2105.0 (s), 2071.2 (m), 1959.9 (m), 1511.9 (m), 1413.6 (s), 1384.2 (m), 1270.8 (s), 1090.2 (m), 1037.7 (m), 1019.1 (m), 964.5 (w), 948.6 (w), 773.8 (vs); ^1H NMR ($CDCl_3$) δ 7.93 (d, *J* = 9, 1H), 7.90 (d, *J* = 9, 1H), 7.38 (apparent t, *J* = 8, 2 H), 6.84 (m, 2 H), 4.46 (t, *J* = 6, 2H), 4.31 (t, *J* = 5, 2H), 3.77 (t, *J* = 6, 2H), 3.72 (t, *J* = 5, 2H); ^{13}C NMR ($CDCl_3$) δ 153.80, 153.59, 126.69, 126.56, 125.27, 125.16, 115.04, 115.00, 106.01, 105.54, 68.02, 67.17, 50.43, 29.26; MS (ESI) 358.4 ($M+Na^+$); HRMS (ESI-TOF) calcd. for $C_{14}H_{15}N_3O_2Br$ $[M+1]^+$: 336.0348; found: 336.0340.

Method B from 9: To a solution of mesylate **9** (0.636 g, 1.81 mmol) in DMF (10 mL) was added KBr (0.502 g, 4.22 mmol) and 18-crown-6 (≈ 5 mg, 0.02 mmol). The mixture was stirred at 45 °C for 23.5 h. The mixture was diluted with EtOAc and water and the organic layer was washed with water (3×), brine, dried (Na_2SO_4), and concentrated to give 0.568 g (94%) of **6** as yellow solid which was identical to the material prepared by Method A above.

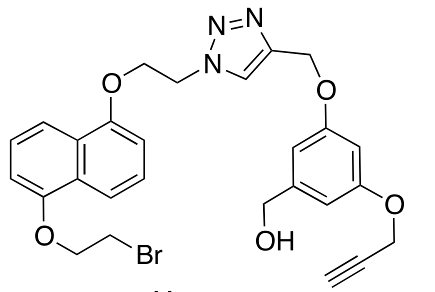

11

4-[3-(Hydroxymethyl)-5-(2-propyn-1-yloxy)phenoxy]methyl]-1-[5-(2-bromoethoxy)-1-naphthyloxy)ethoxy]-1*H*-1,2,3-triazole (11). A solution of **10** (3.220 g, 14.91 mmol), methylene chloride (12 mL), and acetonitrile (3 mL) was degassed by argon bubbling and then $[CH_3CN]_4CuPF_6$ (427 mg, 1.14 mmol), ascorbic acid (2 mL of 0.2 M aqueous solution, 0.4 mmol), and the tris(2-benzimidazolylmethyl)amine ligand (18 mg, 0.044 mmol) were added. This solution was added to a second degassed solution of **6** (1.25 g, 3.72 mmol) in methylene chloride (20 mL), and stirred at room temperature for 3 h. The solution was concentrated and purified by flash chromatography (50% ethyl acetate/petroleum ether, 67% ethyl acetate/petroleum ether, 80% ethyl acetate/petroleum ether, 100% ethyl acetate) to give 866 mg (42%) of **11** as off-white flaky solid. mp 116-118 °C; IR ν 3394.9 (br w), 3287.5 (w), 2929.4 (w), 2871.1 (w), 2121.1 (w), 1685.4 (w), 1594.9 (vs), 1509.2 (m), 1454.7 (m), 1414.5 (s), 1380.6 (m), 1289.3 (m), 1265.8 (s), 1165.3 (s), 1090.0 (m), 1052.1 (s), 971.7 (m), 834.6 (m), 777.0 (s); ^{1}H NMR (DMSO-d_6) δ 8.42 (s, 1H), 7.80 (d, *J* = 9, 1H), 7.65 (d, *J* = 9, 1H), 7.41 (apparent t, *J* = 8, 1H), 7.37 (apparent t, *J* = 8, 1H), 7.03 (d, *J* = 8, 1H), 6.99 (d, *J* = 8, 1H), 6.63 (s, 1H), 6.56 (s, 1H), 6.53 (t, *J* = 2, 1H), 5.22 (t, *J* = 6, 1H), 5.12 (s, 2H), 4.95 (t, *J* = 5, 4H), 4.75 (d, *J* = 2, 2H), 4.56 (t, *J* = 5, 2H), 4.47 (t, *J* = 5, 2H), 4.44 (d, *J* = 6, 2H), 3.93 (t, *J* = 5, 2H), 3.54 (t, *J* = 2, 1H); ^{13}C NMR (DMSO-d_6) δ 159.08, 158.30, 153.28, 145.31, 142.75, 125.93, 125.84, 125.63, 125.55, 125.21, 114.28, 114.14, 106.41, 106.24, 105.37, 100.06, 79.37, 78.17, 68.06, 66.66, 62.79, 61.17, 55.44, 49.25, 31.72; MS (ESI-TOF) 552.1 (M+1, 100), 534.1 (M-H_2O, 86), 205.2 (7); HRMS (ESI-TOF) calcd. for $C_{27}H_{27}N_3O_5Br$ $[M+1]^+$: 552.1134; found: 552.1145.

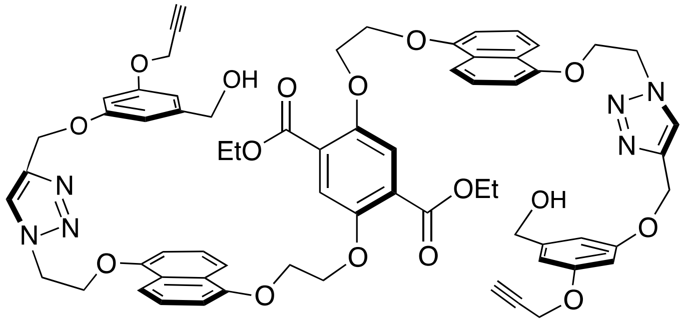

13

Diethyl 2,5-bis[2-[5-[4-[3-(hydroxymethyl)-5-(2-propyn-1-yloxy)phenoxy]methyl]-1*H*-1,2,3-triazol-1-yl]-1-naphthyloxy]ethoxy]-1,4-benzenedicarboxylate (13).

Method A from 11. A mixture of **11** (553 mg, 1.00 mmol), 18-crown-6 (20.4 mg, 0.077 mmol), in DMF (8.5 mL) was added **12** (41.4 mg, 0.164 mmol), and potassium carbonate (232 mg, 1.68 mmol) and the mixture was heated at 85 °C for 200 min. Additional **11** (81 mg, 0.32 mmol) was added and the mixture was heated at 85 °C for 18 h. The mixture was cooled and diluted

with EtOAc (25 mL) and water (25 mL). The organic layer was separated and the aqueous layer was extracted with EtOAc (20 mL), CH_2Cl_2 (3 × 20 mL), and EtOAc (20 mL). The combined organic layers were washed with water (1 × 30 mL), brine (2 × 30 mL), dried (Na_2SO_4), and concentrated to give 527 mg of a brown oil. The oil was dissolved in acetone, CH_2Cl_2, and methanol; silica gel was added, and the solvents were removed under reduced pressure. The silica gel with the crude product absorbed onto it was added to the top of a silica gel column and the product was purified by flash chromatography (1:1 EtOAc/CH_2Cl_2, 1:25:25 MeOH/EtOAc/CH_2Cl_2, 1:20:20 MeOH/EtOAc/CH_2Cl_2) to give 105.2 mg (18%) of **13** as white solid identical to the material prepared by Method B below.

Method B from 14. A mixture of **14** (298 mg, 0.390 mmol), methylene chloride (10 mL), and methanol (4 mL) was heated at ≈ 30˚C until homogeneous. Ascorbic acid (86 mg, 0.48 mmol) was added and the mixture was sonicated until homogenous. Benzyl alcohol **10** (796 mg, 3.68 mmol) was added, the solution was degassed by bubbling with argon, and $[CH_3CN]_4CuPF_6$ (76 mg, 0.20 mmol) was added. The mixture was capped tightly and stirred at room temperature for 240 h. The solution was decanted away from a yellow precipitate and a solid–liquid extraction done with 20% methanol/methylene chloride (10 mL) and acetone (10 mL). The combined solutions were concentrated to a yellow oil which was purified by flash chromatography (1:1 EtOAc/PE, 1:20:20 MeOH/EtOAc/CH_2Cl_2, 1:15:15 MeOH/EtOAc/CH_2Cl_2, 1:10:10 MeOH/EtOAc/CH_2Cl_2, and 1:5:5 MeOH/EtOAc/CH_2Cl_2) to provide 419.1 mg (87%) of **13** as pale yellow solid. mp 156.5-158.0 °C; IR ν 3286.6 (w), 2925.5 (w), 1697.3 (m), 1595.1 (m), 1509.8 (m), 1454.7 (w), 1414.5 (s), 1338.3 (w), 1291.2 (w), 1266.6 (s), 1225.9 (m), 1210.9 (m), 1151.0 (s), 1089.9 (s), 1048.9 (m), 1035.5 (m), 835.1 (w), 767.0 (s); ^{1}H NMR (DMSO-*d6*) δ 8.40 (s, 2H), 7.65 (d, *J* = 9, 2H), 7.62 (d, *J* = 9, 2H), 7.48 (s, 2H), 7.37 (m, 2H), 7.31 (m, 2H), 7.03 (d, *J* = 8, 2H), 6.97 (d, *J* = 8, 2H), 6.61 (s, 2H), 6.55 (s, 2H), 6.51 (s, 2H), 5.18 (t, *J* = 6, 2H), 5.11 (s, 4H), 4.94 (t, *J* = 5, 4H), 4.74 (d, *J* = 2, 4H), 4.54 (t, *J* = 5, 4H), 4.52 (m, 4H), 4.44 (m, 4H), 4.42 (d, *J* = 6, 4H), 4.16 (q, *J* = 7, 4H), 3.54 (apparent s, 2H), 1.11 (t, *J* = 7, 6H); MS (MALDI) 1219.69 (M+Na$^+$, 100), 626.75 (93), 507.63 (97); HRMS (ESI-TOF) calcd. for $C_{66}H_{65}N_6O_{16}$ [M+1]$^+$: 1197.4457; found: 1197.4445.

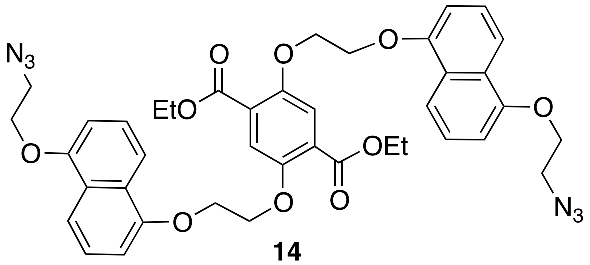

14

Diethyl 2,5-bis[[5-(2-azidoethoxy)-1-naphthyloxy]ethoxy]-1,4-benzene-dicarboxylate (14). A mixture of **6** (212 mg, 0.633 mmol), **12** (73 mg, 0.29 mmol), cesium carbonate (215 mg, 0.660 mmol), and acetone (4 mL) were heated at reflux for 23 h. The mixture was cooled to 5 °C and the yellow precipitate was collected by vacuum filtration, washed with water and diethyl ether, and dried to give 180 mg (82%) of **14** as yellow powder: mp 155-158 °C; IR ν 3320 (w), 2974.0 (w), 2934.1 (w), 2914.3 (w), 2161.2 (w), 2143.2 (w), 2104.3 (m), 2072.8 (m), 1720.7 (m), 1702.1 (m), 1592.7 (m), 1509.5 (m), 1459.6 (w), 1430.2 (w), 1420.1 (s), 1409.9 (s), 1372.2 (m), 1295.0 (m), 1270.3 (s), 1239.6 (m), 1229.2 (m), 1212.3 (s), 1062.1 (s), 1015.8 (w), 969.2 (m), 951.1 (m), 879.6 (w), 775.6 (s), 758.2 (m), 737.2 (w); ^{1}H NMR (DMSO-*d6*) δ 7.76 (d, *J* = 8, 2H), 7.67 (d, *J* =

8, 2H), 7.50 (s, 2H), 7.42 (t, *J* = 8, 2H), 7.33 (t, *J* = 8, 2H), 7.06 (d, *J* = 8, 2H), 6.97 (d, *J* = 8, 2H), 4.53 (m, 4H), 4.47 (m, 4H), 4.33 (t, *J* = 5, 4H), 4.18 (q, *J* = 7, 4H), 3.78 (t, *J* = 5, 4H), 1.13 (t, *J* = 7, 6H); MS (ESI) 765.3 (M+1, 100), 338.3 (32); HRMS (ESI-TOF) calcd. for $C_{40}H_{41}N_6O_{10}$ $[M+1]^+$: 765.2884; found: 765.2853.

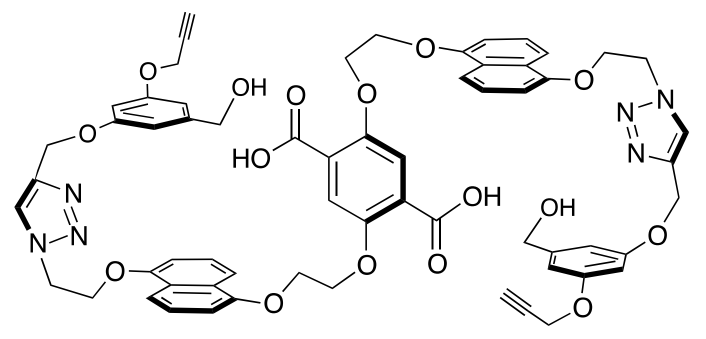

15

2,5-Bis[2-[5-[4-[3-(hydroxymethyl)-5-(2-propyn-1-yloxy)phenoxy]methyl]-1*H*-1,2,3-triazol-1-yl]-1-naphthyloxy]ethoxy]-1,4-benzenedicarboxylic acid (15). To a solution of KOH (80 mg, 1.4 mmol) in water (2.0 mL) was added **13** (43 mg, 36 mmol) in THF (2.0 mL) and ethanol (2.0 mL). The heterogeneous mixture was stirred at 95°C for 95 h. Upon cooling to room temperature, 1 M hydrochloric acid (1 mL) was added to the solution. The mixture was partitioned between saturated aqueous NH_4Cl and methylene chloride. The aqueous layer was extracted with methylene chloride (3 × 2 mL). The combined organic layers were diluted with methanol (2 mL) and washed with brine, dried (Na_2SO_4), and concentrated to give 32 mg (78%) of **15** as off-white solid. mp 190-191 °C; IR ν 3161.0 (w), 3054.4 (w), 2931.1 (w), 1723.0 (m), 1594.0 (s), 1508.6 (m), 1453.9 (m), 1413.4 (s), 1339.7 (w), 1291.7 (w), 1264.2 (s), 1210.9 (w), 1157.5 (m), 1089.4 (m), 1049.5 (m), 963.7(w), 832.0 (w), 777.0 (m); ^1H NMR (DMSO-d_6) δ 12.99 (br s, 2H), 8.40 (s, 2H), 7.71 (d, *J* = 9, 2H), 7.62 (d, *J* = 9, 2H), 7.51 (s, 2H), 7.37 (m, 2H), 7.33 (m, 2H), 7.04 (d, *J* = 8, 2H), 6.98 (d, *J* = 7, 2H), 6.61 (d, *J* = 1, 2H), 6.54 (s, 2H), 6.51 (m, 2H), 5.18 (t, *J* = 5, 2H), 5.11 (s, 4H), 4.94 (t, *J* = 5, 4H), 4.74 (d, *J* = 2, 4H), 4.54 (t, *J* = 5, 4H), 4.52 (m, 4H), 4.44 (m, 4H), 4.42 (d, *J* = 5, 4H), 3.54 (t, *J* = 2, 2H); MS (ESI-TOF) 1141.4 (M+1); HRMS (ESI-TOF) calcd. for $C_{62}H_{57}N_6O_{16}$ $[M+1]^+$: 1141.3831; found: 1141.3870.

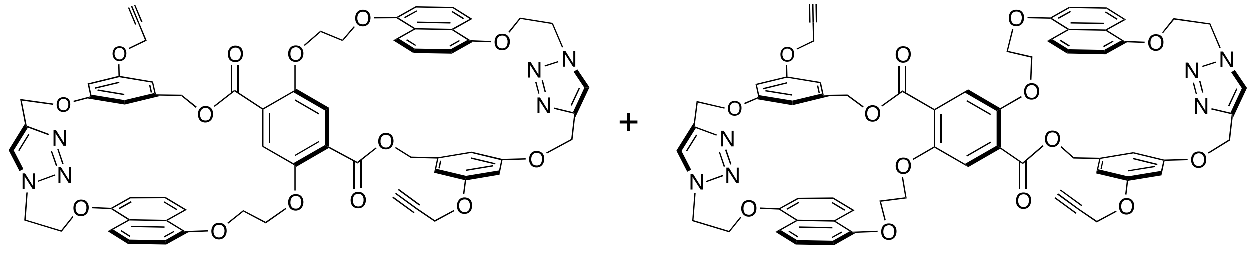

1 meta isomer **1** ortho isomer

Bismacrocyclic host (1). To a solution of 2-methyl-6-nitrobenzoic anhydride (4.2 mg, 0.13 mmol), and 4-dimethylaminopyridine (31 mg, 0.25 mmol) in acetone (20 mL) was added a solution of **14** (45 mg, 0.039 mmol) in acetone (180 mL) dropwise over 2 h. After the addition was complete, the mixture was stirred at room temperature for 24 h. The solution was concentrated to ≈ 5 mL and cooled in a refrigerator at 5 °C overnight. The solid precipitate was

collected on a fine porosity glass frit and washed well with methanol (2 × 4 mL) and diethyl ether (1 × 4 mL). The solid was then suspended in a boiling solvent system of 1:1:1 acetone/methanol/$CHCl_3$ (30 mL). The insoluble precipitate was collected on a fine porosity glass frit and washed with acetone, warm methanol, 20% methanol/methylene chloride, and diethyl ether to give 12.1 mg (28%) of **1** as gray-white solid. mp 216 °C (dec.); IR ν 2912 (w), 1719.8 (w), 1595.4 (s), 1508.2 (m), 1458.9 (w), 1413.7 (s), 1363.9 (w), 1292.2 (w), 1264.4 (s), 1230.2 (m), 1211.2 (m), 1196.0 (w), 1166.3 (m), 1154.8 (m), 1109.9 (w), 1089.0 (s), 1055.8 (s), 1026.1 (w), 963.4(w), 775.7 (m); ^{1}H NMR (DMSO-*d*$_6$) δ 8.15 (s, 2H), 7.41 (s, 2H), 7.41 (d, *J* = 8, 2H), 7.34 (d, *J* = 9, 2H), 7.15 (apparent t, *J* = 8, 2H), 7.06 (apparent t, *J* = 8, 2H), 7.02 (d, *J* = 8, 2H), 6.72 (d, *J* = 7, 2H), 6.51 (s, 2H), 6.38 (s, 2H), 6.00 (s, 2H), 5.11 (s, 4H), 4.90 (m, 4H), 4.71 (d, *J* = 2, 4H), 4.59 (s, 4H), 4.54 (s, 4H), 4.48 (br s, 12H), 3.59 (s, 2H); MS (MALDI) 1105.8 (M+1, 100), 1127.9 (M+Na$^+$, 67), 1143.9 (M+K$^+$, 34); HRMS (ESI-TOF) calcd. for $C_{62}H_{53}N_6O_{14}$ [M+1]$^+$: 1105.3620; found: 1105.3595.

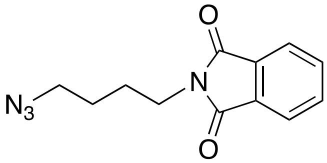

1-Phthalimidyl-4-azidobutane. To a solution of **17** (1.54 g, 11.6 mmol) in DMF (30 mL) was added potassium phthalimide (2.22 g, 12.0 mmol) and KI (0.210 g, 1.27 mmol). The mixture was stirred for 4 days at 70 °C. On the fifth day the mixture was worked up by adding ether (40 mL) and washing with water (2 × 40 mL) and brine (1 × 40 mL). The ethereal phase was isolated, dried with Na_2SO_4, and concentrated under reduced pressure to give 1.68 g (59%) of 1-phthalimidyl-4-azidobutane as yellow oil. All spectroscopic data matched the values reported in literature.[9]

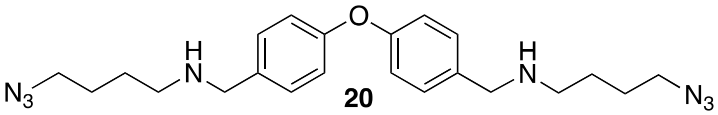

20

Bis[4-(*N*-4-azidobutyl)aminomethylphenyl] ether (20). A solution of **19** (0.182 g, 0.807 mmol) and **18** (0.317 g, 2.83 mmol) in toluene (40 mL) was heated at reflux under a Dean–Stark trap. Imine formation was monitored by IR spectroscopy and the reaction was complete after 24 h. The mixture was cooled to rt, and $NaBH_4$ (0.153 g, 4.04 mmol) in EtOH (5 mL) was added. The mixture was stirred at ambient temperature and then heated at reflux. After 5 days the solution was concentrated under reduced pressure. The crude brown residue was partitioned between aqueous NaOH (1.5 M, 30 mL) and CH_2Cl_2 (30 mL). The aqueous layer was extracted with CH_2Cl_2 (2x). The organic layers were combined and concentrated, giving a crude brown oil. The product was purified by flash chromatography (7% MeOH/CH_2Cl_2) to give 48.6 mg (14%) of **20** as an oil. IR ν 2933 (m), 2863 (w), 2093 (s), 1601 (m), 1500 (s), 1237 (s), 1167 (m), 1115 (m), 1015 (w), 874 (m), 834 (m); ^{1}H NMR (acetone-*d*$_6$) 7.356 (d, *J* = 9, 4H), 6.933 (d, *J* = 9, 4H), 3.754 (s, 4H), 3.346 (t, *J* = 7, 4H), 2.639 (t, *J* = 7, 4H), 1.680 (m, 4H), 1.592 (m, 4H); MS (ESI) 423.3 (M+1, 32), 212.1 ([M+2]/2, 70); HRMS (ESI-TOF) calcd. for $C_{22}H_{31}N_8O$ [M+1]$^+$: 423.2621; found: 423.2636.

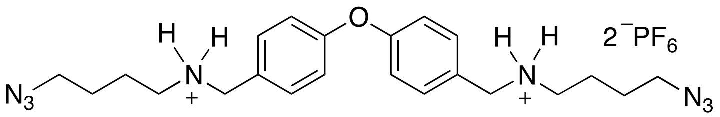

2

Bis[4-(*N*-4-azidobutyl)ammoniummethylphenyl] ether bis(hexafluorophosphate) (2). Diamine **20** (0.153 g, 0.362 mmol) was dissolved in a minimum volume of CH_2Cl_2. The mixture was stirred while HCl in dioxane (0.40 mL, 1.6 mmol) was added dropwise. The chloride salt precipitated and the solvent was removed under reduced pressure. This material was dissolved a minimum volume of 1:1 EtOH/water and a solution of NH_4PF_6 (0.725 g, 4.45 mmol) in a minimum volume of water was added with stirring. The mixture was concentrated to a white solid, which was collected by vacuum filtration and washed with cold water to give 186 mg (72%) of **2** white solid. mp 213-215 °C; IR ν 3264 (m), 3240 (w), 2938 (w), 2099 (s), 1508 (m), 1605 (w), 1583 (m), 1421 (m), 1257 (s), 1174 (w), 894 (m), 845 (vs), 767 (s); ^{1}H NMR (acetone-d_6) δ 8.194 (br s, 4H), 7.656 (d, *J* = 9, 4H), 7.118 (d, *J* = 9, 4H), 4.613 (s, 4H), 3.498 (t, *J* = 8, 4H), 3.441 (t, *J* = 7, 4H), 2.167 (m, 4H), 1.759 (m, 4H). ^{13}C NMR (CD_3OD) δ 159.23, 133.03, 127.77, 120.49, 51.74, 51.71, 48.04, 26.8, 24.52; MS (ESI-TOF) 569.2 (M – PF_6^-, 39), 238.1 (76), 212.1 (M – $[PF_6^-]_2$, 100); HRMS (ESI-TOF) calcd. for $C_{22}H_{32}N_8OF_6P$ [M – PF_6^-]$^+$: 569.2341; found: 569.2325.

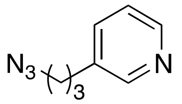

22

3-(3-Azidopropyl)pyridine (22). A stirred solution of **21** (5.192 g, 37.90 mmol) and triethyl amine (6.8 mL, 49 mmol) in DMF (25 mL) was cooled in an ice–water bath while methanesulfonyl chloride (3.8 mL, 49 mmol) was added dropwise over 15 min. The mixture was warmed to ambient temperature and stirred for 135 min. Methanol (1 mL) was added and, after stirring for 25 min, sodium azide (3.33 g, 51.2 mmol) was added. The resulting mixture was stirred for an additional 2.5 d. The mixture was partitioned between diethyl ether (50 mL) and water (75 mL). The aqueous layer was extracted with diethyl ether (2 × 25) and the combined organic layers were washed with water, dilute aqueous ammonium chloride, water, and brine (2×), dried (Na_2SO_4), and concentrated under reduced pressure to give 5.042 g (82%) of **22** as copper-colored liquid that was pure by TLC and NMR: IR ν 3375.6(br w), 2936.6 (w), 2866.6(w), 2091.7 (s), 1576.4 (w), 1479.0 (w), 1452.0 (w), 1423.3 (m), 1348.3 (w), 1257.2 (m), 1191.3 (w), 1044.9 (w), 1027.2 (w), 825.2 (w), 791.3 (m), 712.2 (s), 629.7 (m); ^{1}H NMR ($CDCl_3$) δ 8.47 (m, 2H), 7.51 (dt, *J* = 8, *J* = 2.0, 1H), 7.23 (ddd, *J* = 8, *J* = 5, *J* = 1, 1H), 3.32 (t, *J* = 7, 2H), 2.72 (t, *J* = 8, 2H), 1.92 (m, 2H); ^{13}C NMR ($CDCl_3$) δ 149.69, 147.49, 135.95, 135.65, 123.21, 50.23, 29.92, 29.68; MS (ESI) 162.7 (M+1). All spectroscopic data matched the values reported in literature.[10]

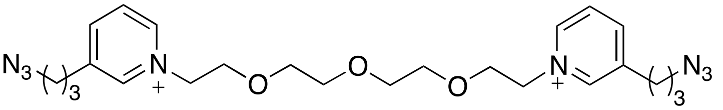

3 $2\ \bar{P}F_6$

Bis[2-[3-(3-Azidopropyl)pyridinium]ethoxy]ethyl] ether bis(hexafluorophosphate) (3). A solution of dibromide **23** (0.6737 g, 2.105 mmol) and **22** (0.692 g, 4.27 mmol) in DMF (5 mL) was

stirred at ambient temperature for 14 d. The solution was concentrated under reduced pressure to give a brown oil. To the oil was added a solution of NH_4PF_6 (4.940 g, 30.3 mmol) in a minimal volume of water. Methylene chloride was then added and the organic layer was separated and concentrated under reduced pressure. The resulting brown oil was purified on a reversed-phase preparatory TLC plate (1:5:94 ammonium hydroxide/CH_3OH/CH_2Cl_2). The UV-active band with the highest R_f value was isolated to give 205.4 mg (21%) of **3** as dark yellow oil. IR ν 2926.0 (s), 2855.1 (m), 2102.2 (s), 1505.8 (m), 1464.8 (m), 1353.0 (w), 1256.1 (m), 1118.2 (m), 834.9 (vs), 689.6 (s); ^{1}H NMR (Acetone-d_6) δ 9.03 (s, 2H), 8.95 (d, J = 6, 2H), 8.65 (d, J = 8, 2H), 8.28 (m, 2H), 4.95 (t, J = 5, 4H), 4.10 (t, J = 5, 4H), 3.64 (m, 4H), 3.53 (m, 4H), 3.48 (t, J = 7, 4H), 3.05 (t, J = 8, 4H), 2.06 (m, 4H); MS (ESI) 629.3 (M – PF_6^-, 100), 214.1 (M – $[PF_6^-]_2$, 83); HRMS (ESI-TOF) calcd. for $C_{24}H_{36}N_8O_3F_6P$ [M – PF_6^-]$^+$: 629.2552; found: 629.2535.

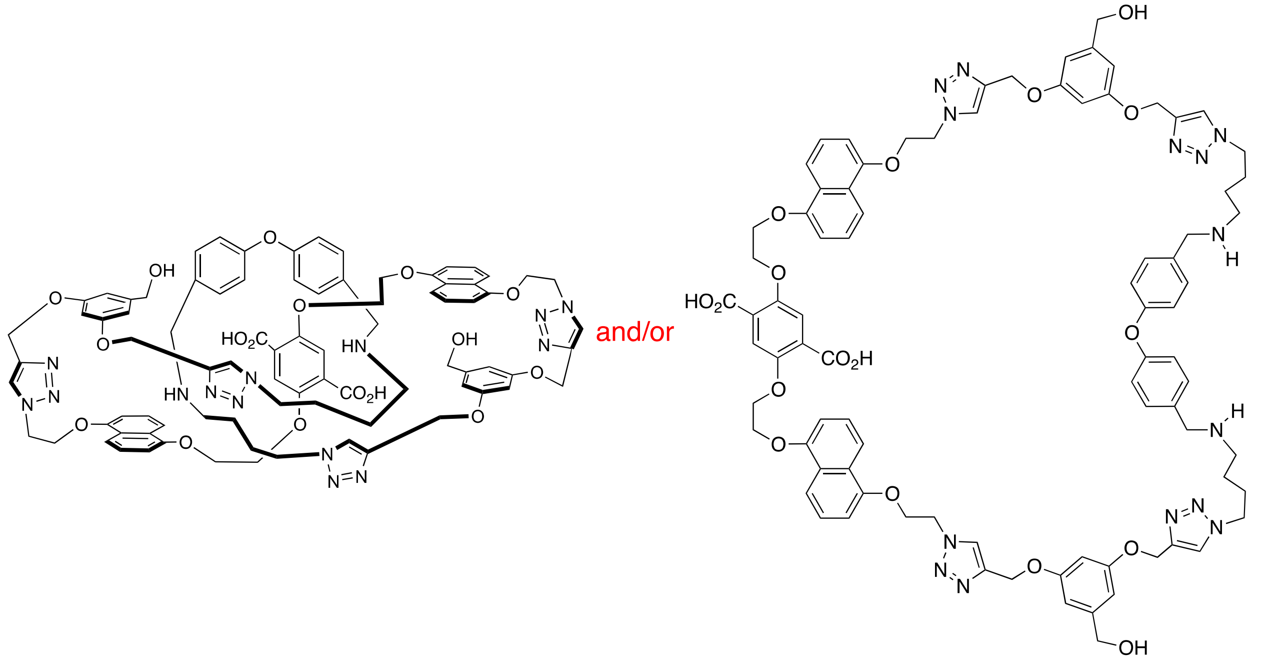

TLC reaction sequence using host 1 and guest 2. A mixture of **1** (2.6 mg, 2.4 μmol), **2** (1.7 mg, 2.4 μmol), $[CH_3CN]_4CuPF_6$ (1.4 mg, 3.8 μmol), CH_3CN (1 mL), CH_2Cl_2 (1 mL), and CH_3OH (0.1 mL) was stirred at ambient temperature for 20 h. Ascorbic acid (5.3 mg, 30. μmol) was added and the mixture was stirred for 5 more days. The solvent was then removed under reduced pressure. The solid was dissolved in a solvent mixture of H_2O (0.2 mL), THF (0.3 mL), and EtOH (0.3 mL) and KOH (10 mg, 179 μmol) was added. The mixture was heated at reflux overnight and then concentrated to a small volume. The pH of the solution was adjusted to neutral by the addition of 1 M HCl (0.15 mL) and the precipitate was collected and dried to give 5.6 mg (124%) of the crude product as a tan solid. The excess mass was assumed to be from cation sequestration and PF_6^- anions which was consistent with the IR and mass spectral data. IR ν 2957.5 (m), 2918.3 (s), 2849.5 (M), 1769.9 (w), 1733.5 (m), 1716.0 (m), 1698.6 (m), 1683.5 (m), 1596.1 (w), 1560.1 (w), 1456.1 (w), 1415.7 (m), 1394.5 (w), 1362.6 (w), 1265.8 (m), 1245.3 (m), 1210.1 (m), 1152.1 (m), 1089.8 (m), 1066.5 (m), 1053.8 (m), 1021.2 (w), 844.6 (vs), 713.8 (w), 669.3 (w), 619.0 (w), 560.0 (m); ^{1}H NMR (DMSO-d_6, 45 °C) δ 8.85 (br s, 4H), 8.38 (s, 2H), 8.19 (s, 2H), 7.71 (app s, 2H), 7.60

(app s, 2H), 7.50 (app s, 6H), 7.34 (app s, 4H), 7.03 (m, 10H), 6.57 (app s, 6H), 5.10 (app s, 6H), 4.92 (app s, 4H), 4.55-4.40 (m, 16H), 4.09 (app s, 4H), 2.95 (app s, 4H), 1.90 (app s, 4H), 1.61 (app s, 4H), 1.22 (app s, 4H), 0.85 (app s, 4H); MS (MALDI) 1563.9 ($M+H^+$, 88), 1584.9 ($M+Na^+$, 28), 1601.9 ($M+K^+$, 52), 1626.8 ($M+Cu^+$, 42), 425.1 (100).

References

[1] Tookmanian, E. M.; Phillips-Piro, C. M.; Fenlon, E. E.; Brewer, S. H. *Chem-Eur J* **2015**, *21*, 19096-19103.

[2] Dyer, E.; Scott, H. *J Am Chem Soc* **1957**, *79*, 672-675.

[3] Antoni, P.; Nystrom, D.; Hawker, C. J.; Hult,A.; Malkoch, M. *Chem Commun* **2007**, 2249-2251.

[4] Yao, L.; Smith, B. T.; Aube, J. *J Org Chem* **2004**, *69*, 1720-1722.

[5] Cunningham, C. W.; Mukhopadhyay, A.; Lushington, G. H.; Blagg, B. S. J.; Prisinzano, T. E.; Krise, J. P. *Mol Pharmaceut* **2010**, *7*, 1301-1310.

[6] Carboni, B.; Benalil, A.; Vaultier, M. *J Org Chem* **1993**, *58*, 3736-3741.

[7] Yeager, G. W.; Schissel, D. N. *Synthesis-Stuttgart* **1991**, 63-68.

[8] Breyholz, H. J.; Wagner, S.; Faust, A.; Riemann, B.; Holtke, C.; Hermann, S.; Schober, O.; Schafers, M.; Kopka, K. *ChemMedChem* **2010**, *5*, 777-789.

[9] Varghese, S.; Gupta, D.; Baran, T.; Jiemjit, A.; Gore, S. D.; Casero, R. A.; Woster, P. M. *J Med Chem* **2005**, *48*, 6350-6365.

[10] Freitas, L. B. D.; Borgati, T. F.; de Freitas, R. P.; Ruiz, A. L. T. G.; Marchetti, G. M.; de Carvalho, J. E.; da Cunha, E. F. F.; Ramalho, T. C.; Alves, R. B. *Eur J Med Chem* **2014**, *84*, 595-604.

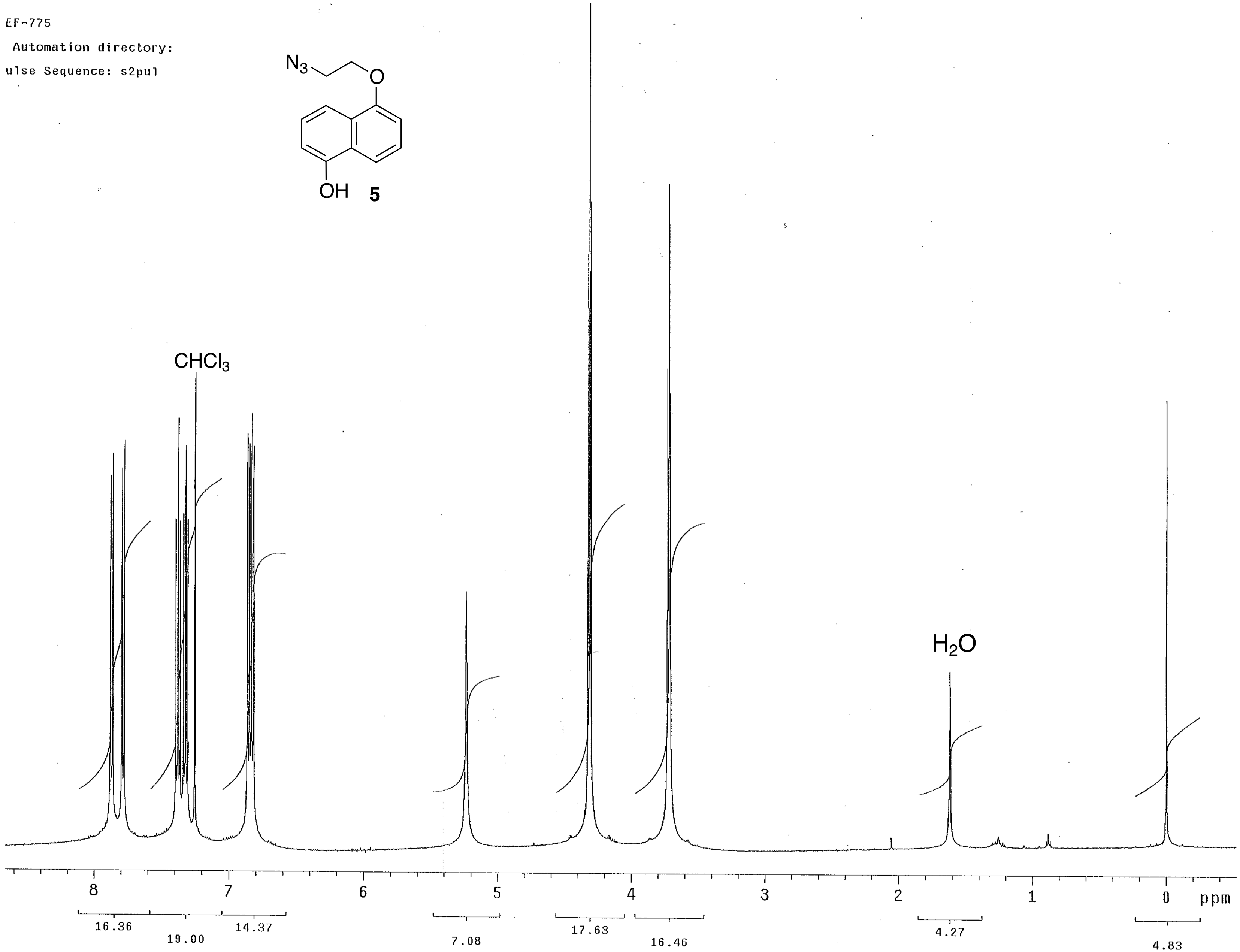
EF-775
Automation directory:
ulse Sequence: s2pul
N_3
O
OH
5
$CHCl_3$
H_2O
8
7
6
5
4
3
2
1
0
ppm
16.36
19.00
14.37
7.08
17.63
16.46
4.27
4.83

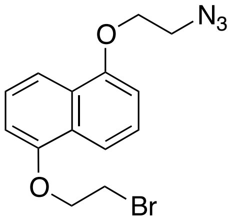
O
N3
O
Br

6

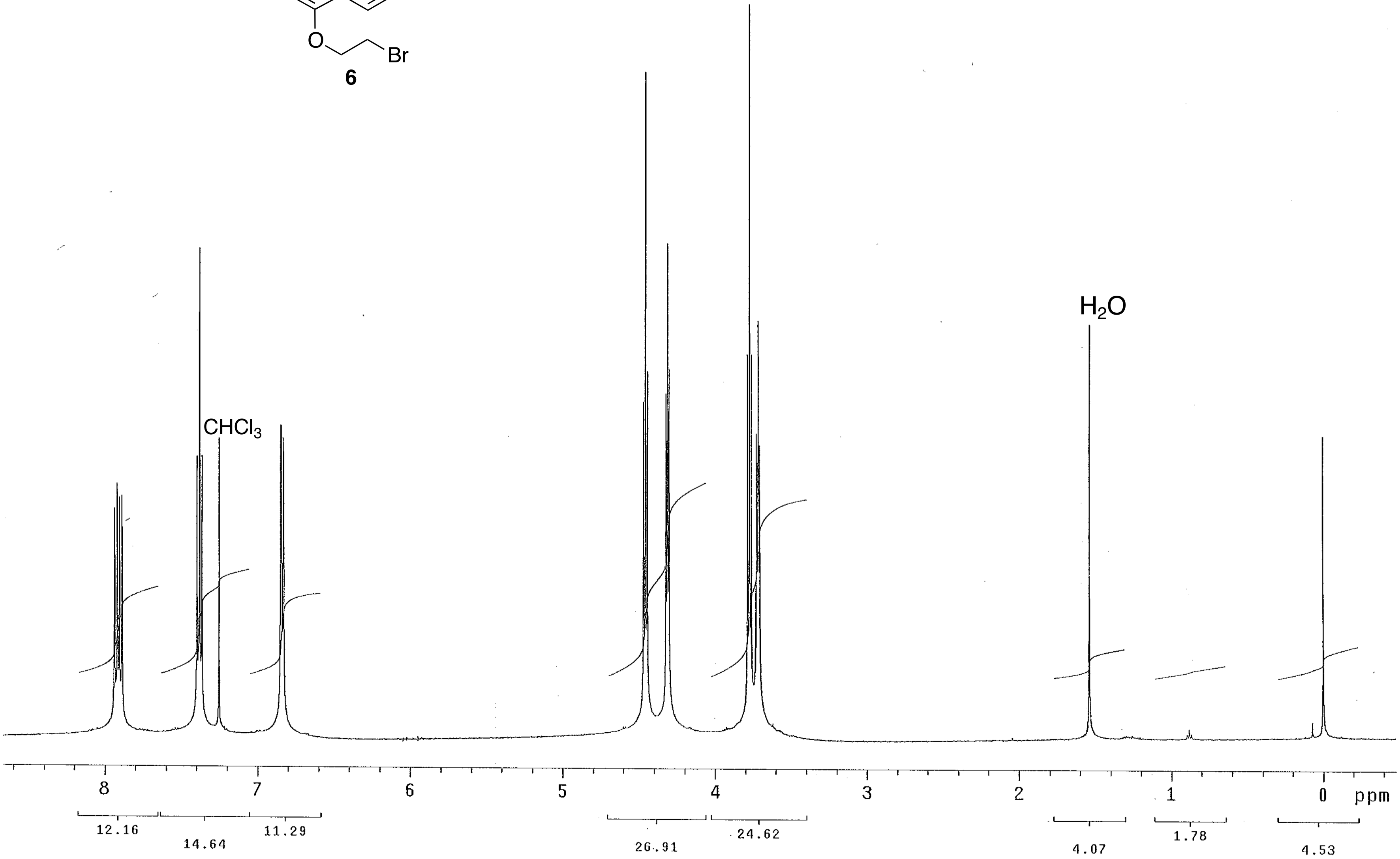
CHCl3
H2O
8
7
6
5
4
3
2
1
0
ppm
12.16
14.64
11.29
26.91
24.62
4.07
1.78
4.53

EEF785
Automation directory:
Pulse Sequence: s2pul

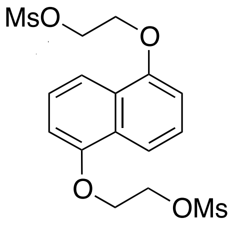

8

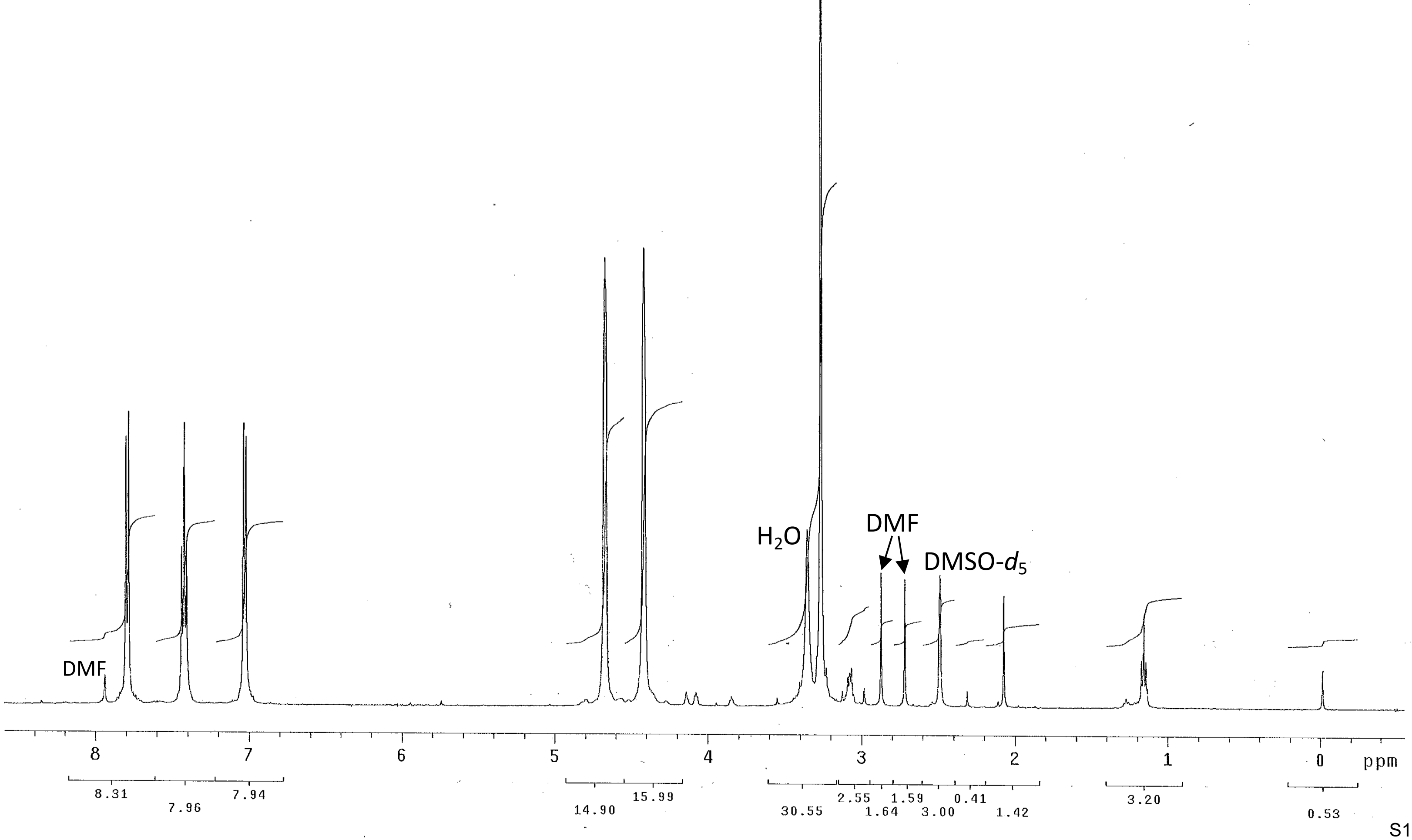

EEF873C

Automation directory:

Pulse Sequence: s2pul

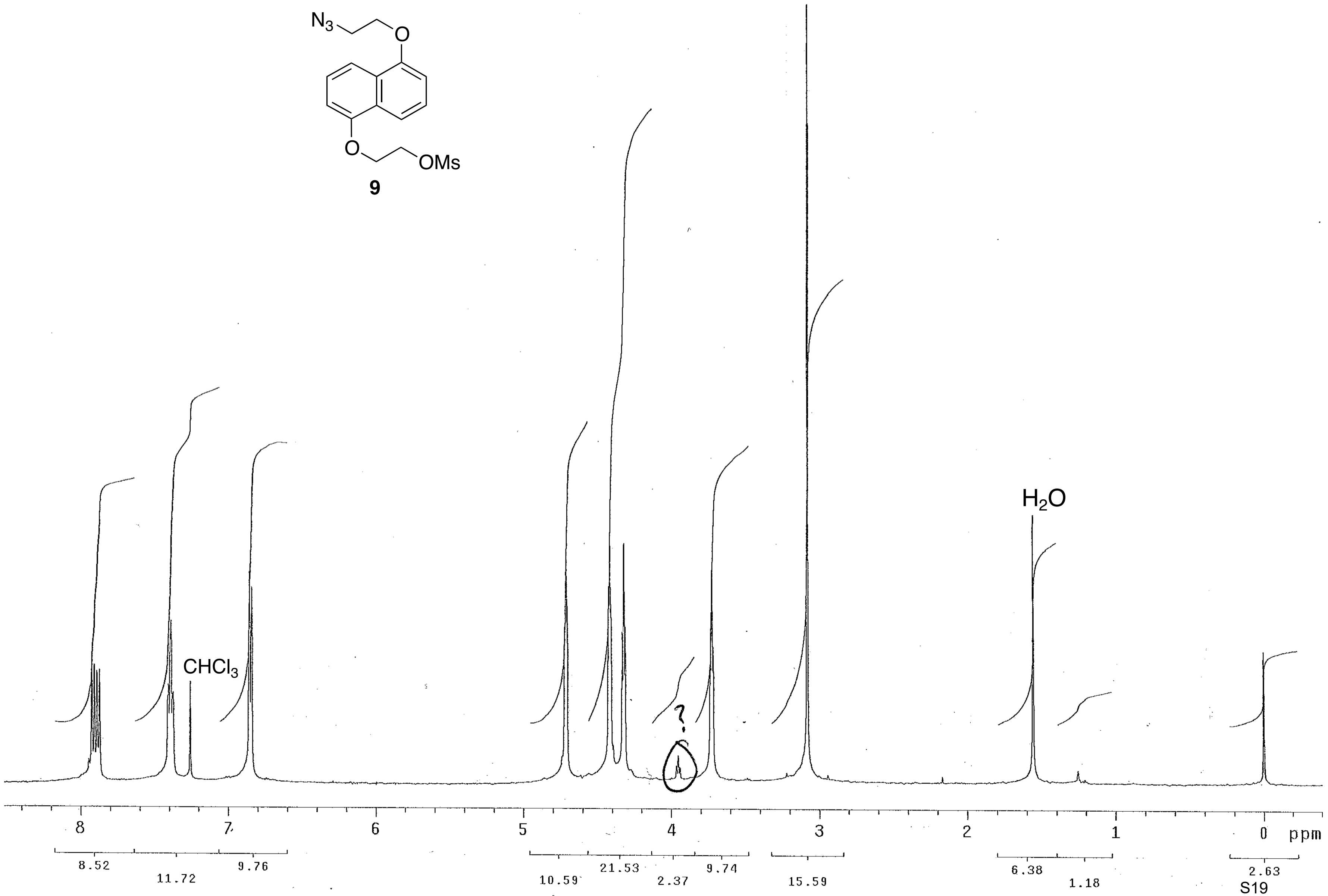

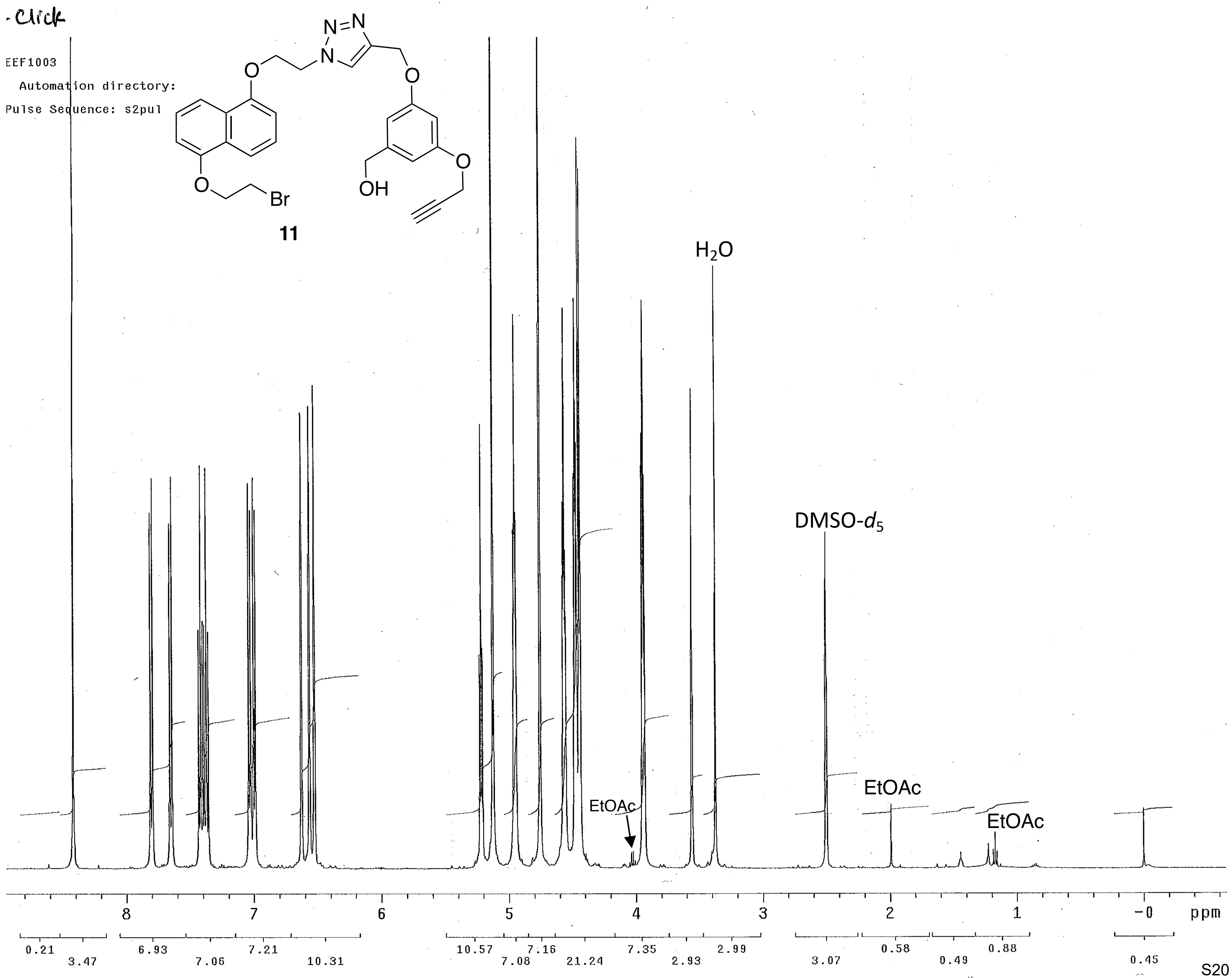
-Click
EEF1003
Automation directory:
Pulse Sequence: s2pul
11
H2O
DMSO-d5
EtOAc
EtOAc
EtOAc
8
7
6
5
4
3
2
1
-0
ppm
0.21
3.47
6.93
7.06
7.21
10.31
10.57
7.08
7.16
21.24
7.35
2.93
2.99
3.07
0.58
0.49
0.88
0.45

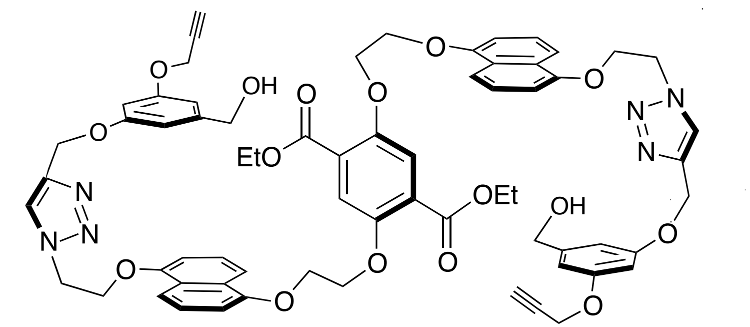

13

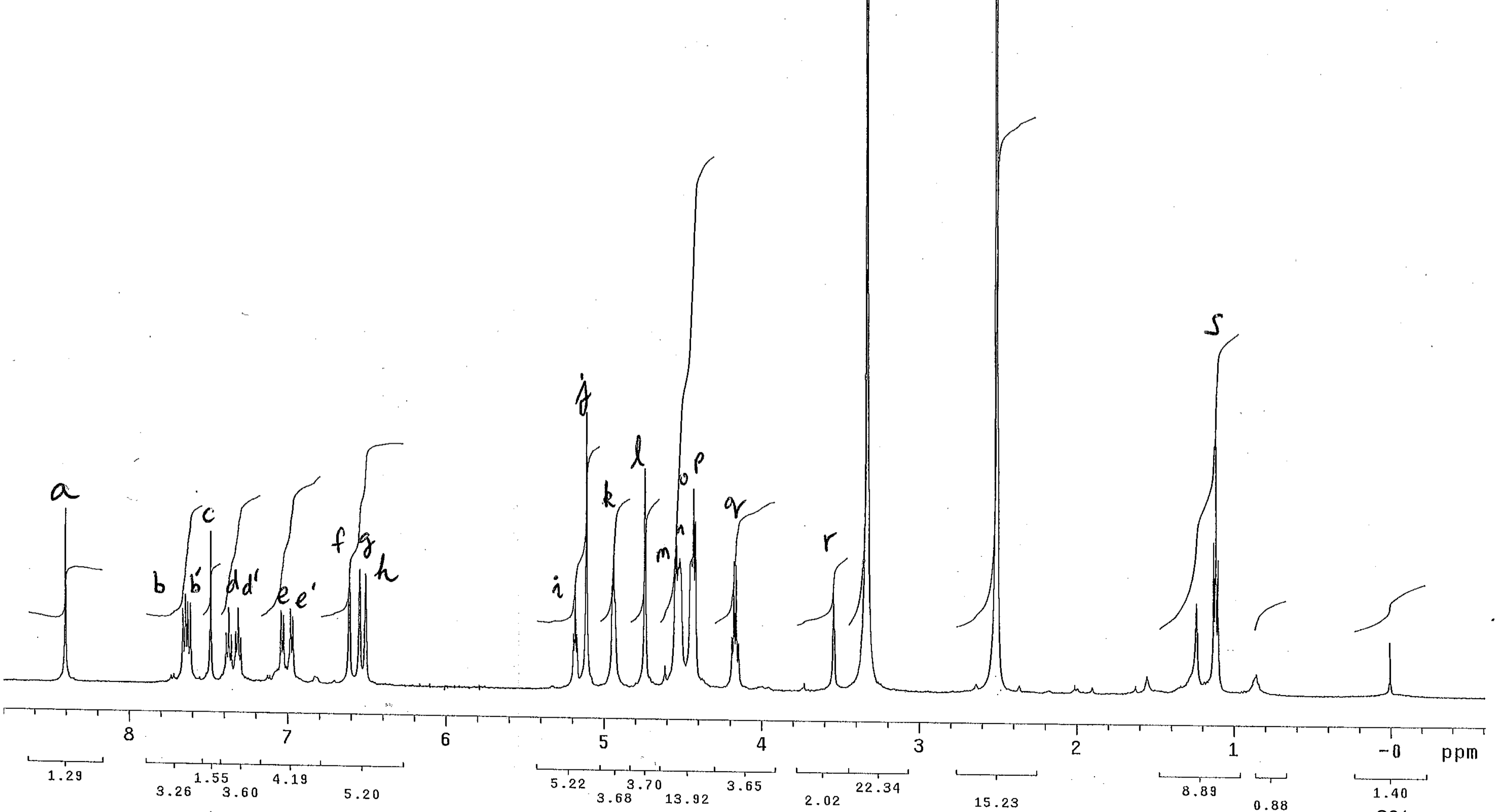
H2O
DMSO-d5
a
b
b'
c
d d'
e e'
f g h
i
j
k
l
m n
o p
q
r
s
8
7
6
5
4
3
2
1
-0
ppm
1.29
3.26
1.55
3.60
4.19
5.20
5.22
3.68
3.70
13.92
3.65
2.02
22.34
15.23
8.89
0.88
1.40

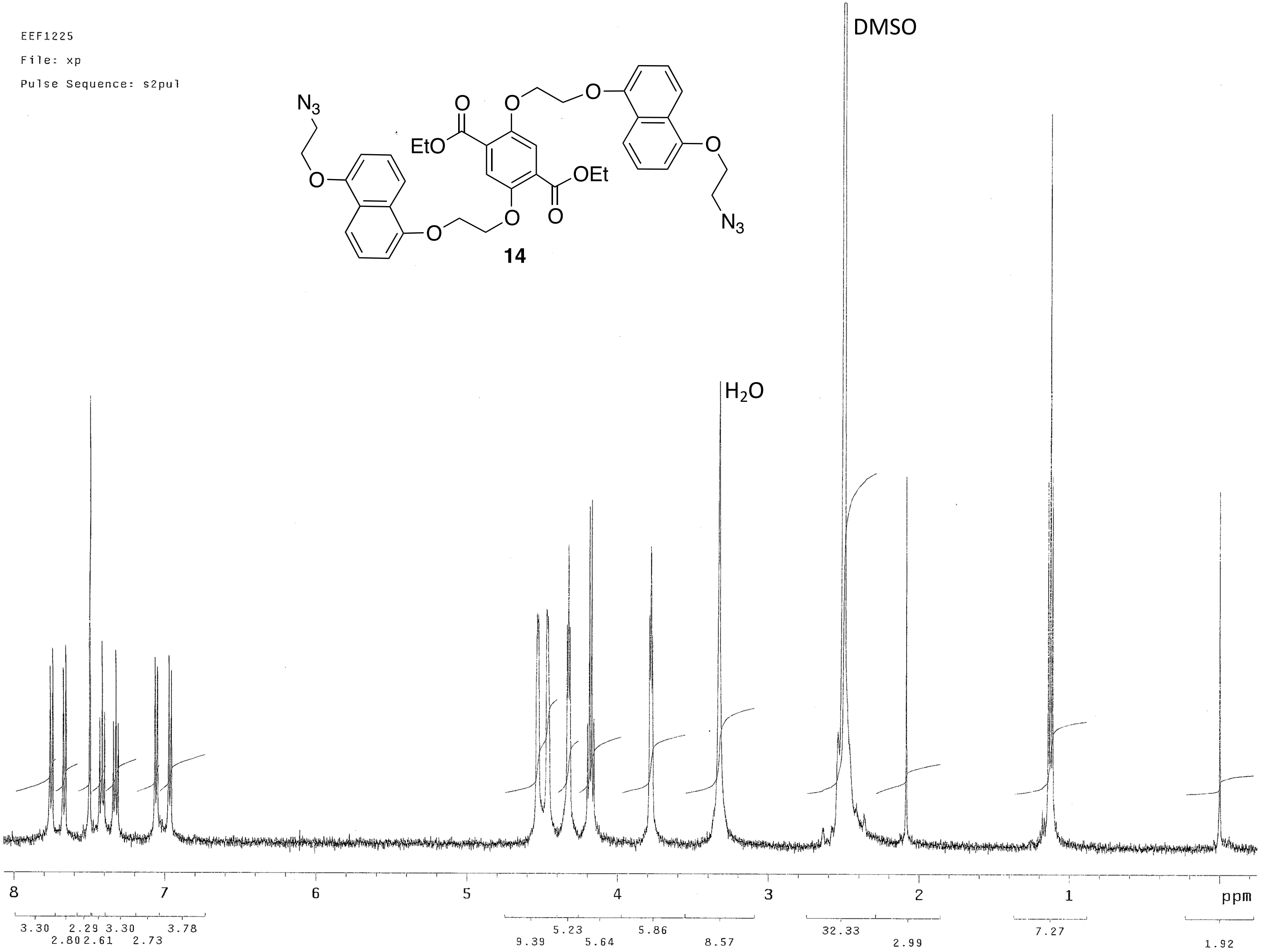
EEF1225
File: xp
Pulse Sequence: s2pul
N3
EtO
OEt
N3
14
DMSO
H2O
8
7
6
5
4
3
2
1
ppm
3.30 2.29 3.30 3.78
2.80 2.61 2.73
5.23 5.86
9.39 5.64 8.57
32.33
2.99
7.27
1.92

EEF1001
Automation directory:
Pulse Sequence: s2pul

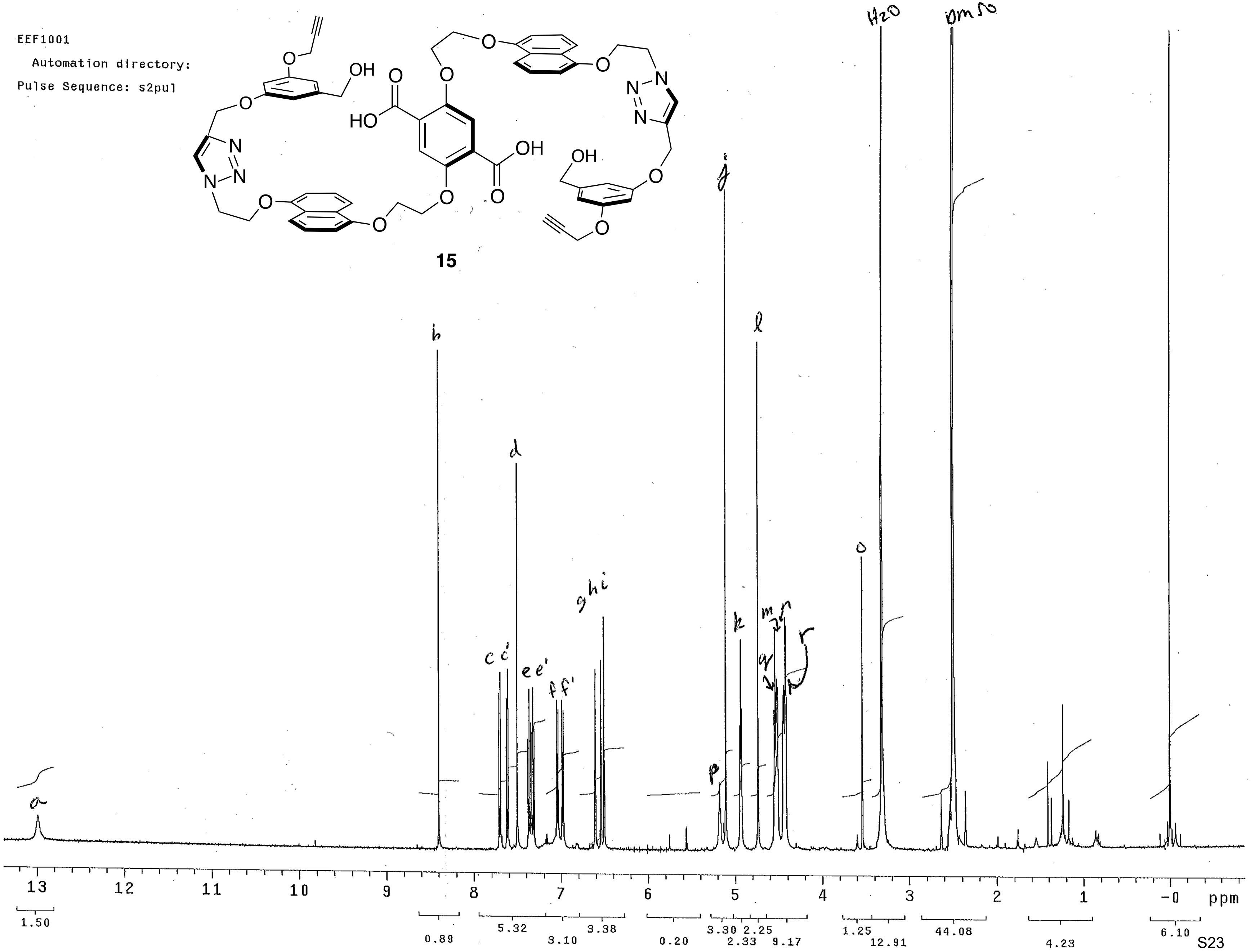

15

S23

EEF1039P

Automation directory:

Pulse Sequence: s2pul

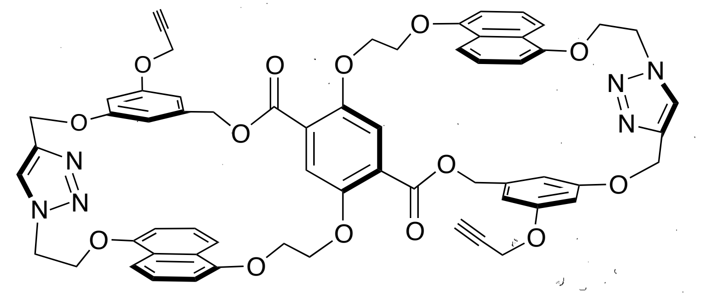

1 meta isomer

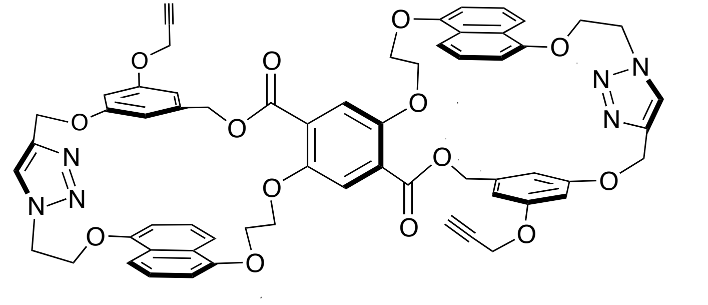

1 ortho isomer

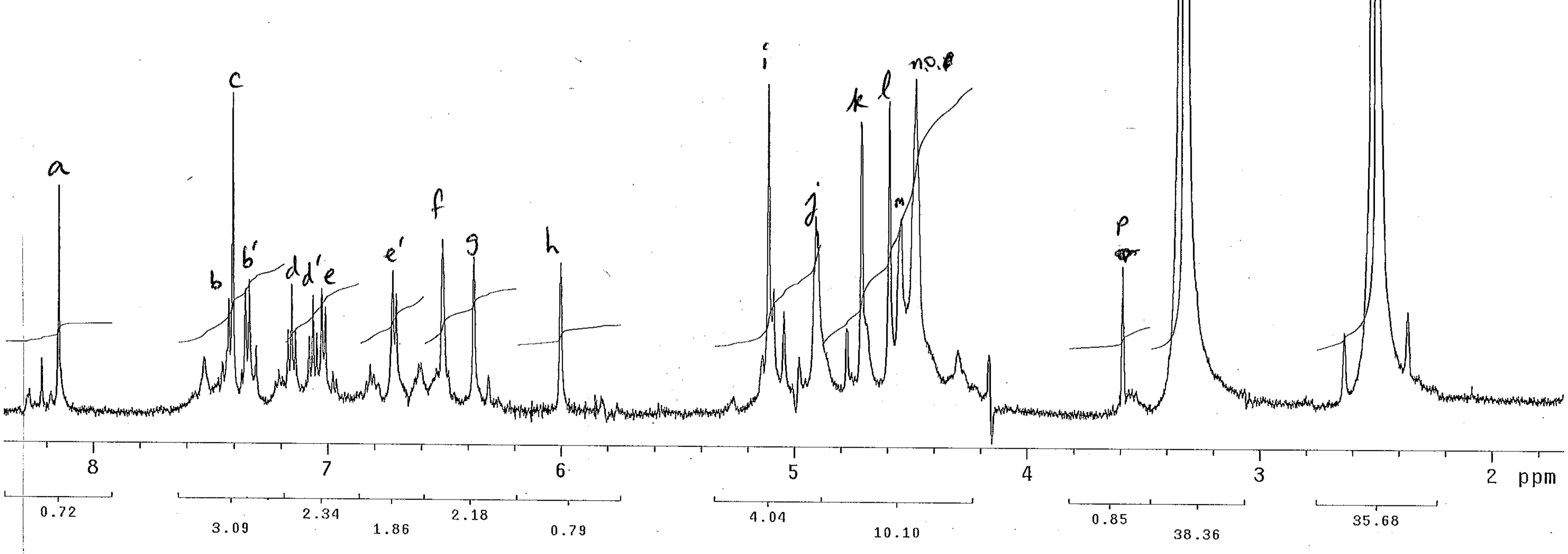

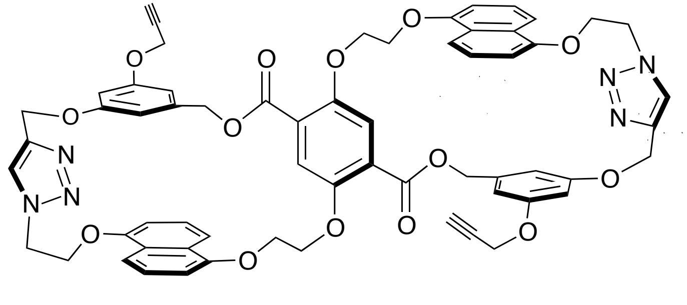

1 meta isomer

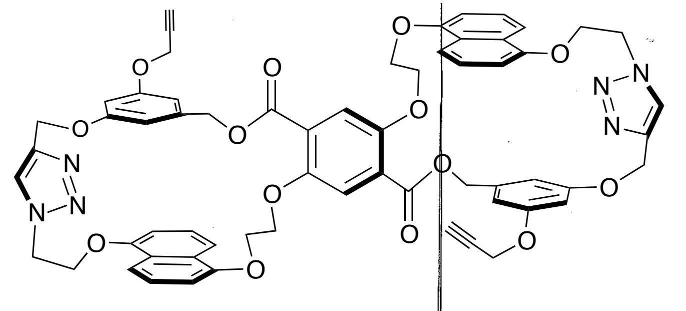

1 ortho isomer

expansion of aromatic region

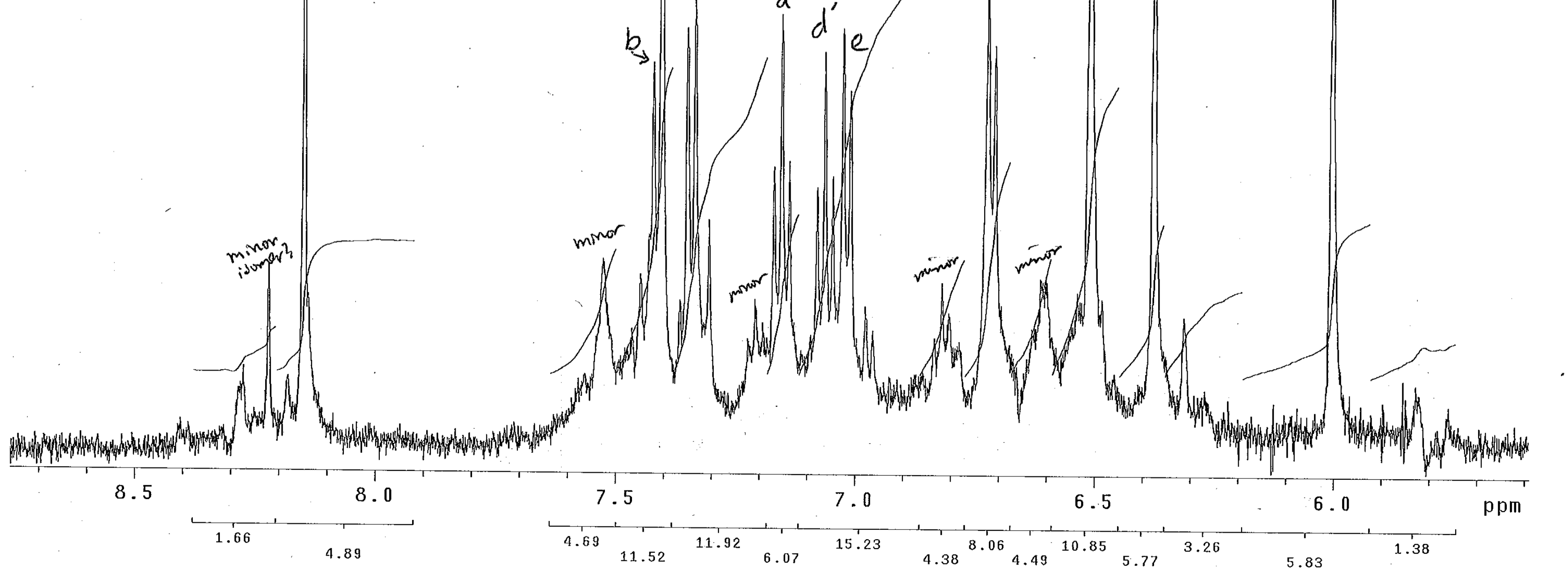

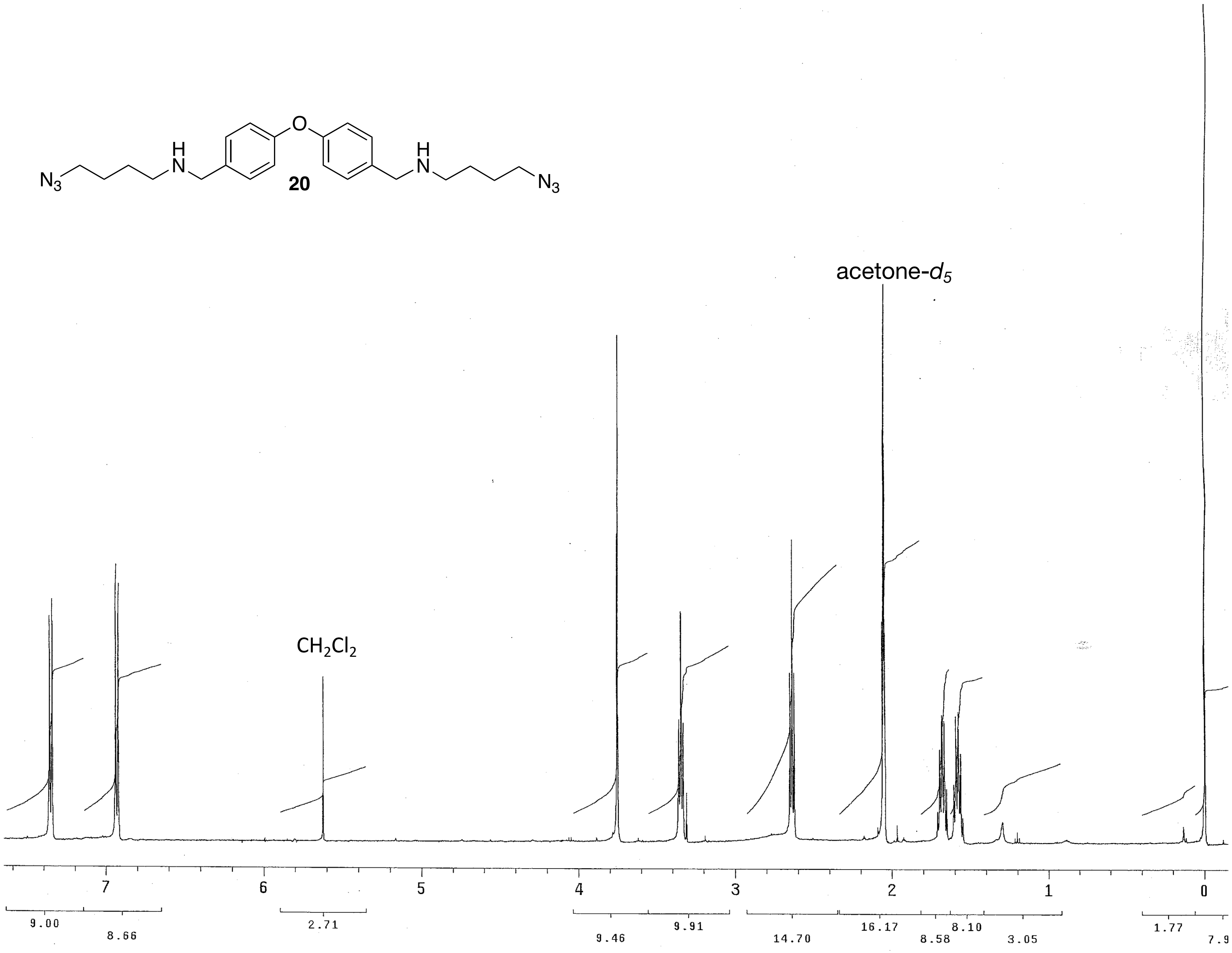

N3
H N
O
20
H N
N3
acetone-d5
CH2Cl2
7
6
5
4
3
2
1
0
9.00
8.66
2.71
9.46
9.91
14.70
16.17
8.58
8.10
3.05
1.77
7.9

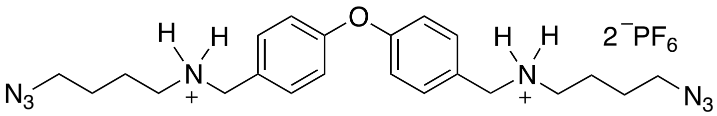
H
H
N
+
O
H
H
N
+
N_3
N_3
2 $^-PF_6$

2

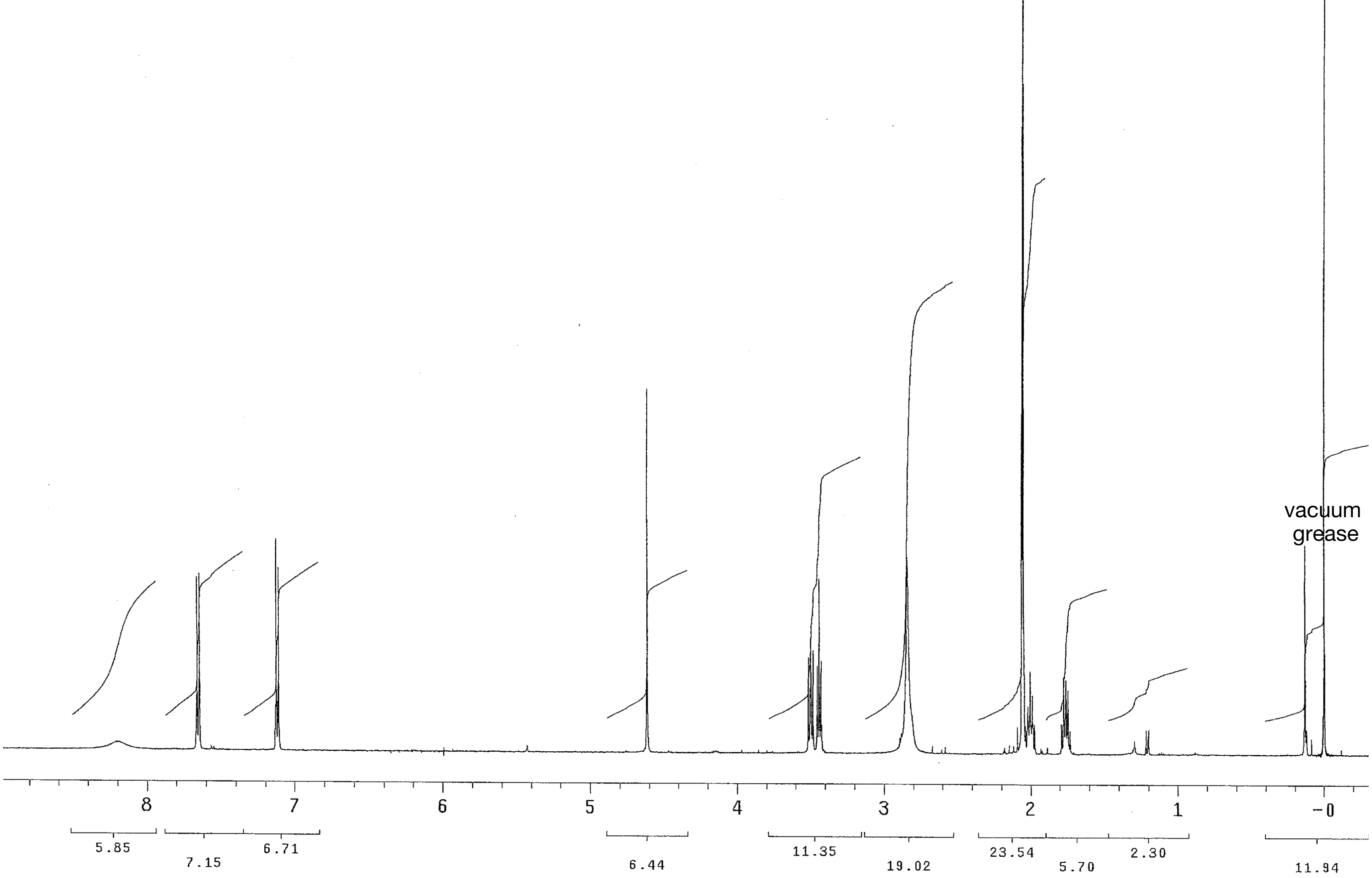
SDL2
Au
Puls
acetone-d_5
vacuum grease
8
7
6
5
4
3
2
1
-0
5.85
7.15
6.71
6.44
11.35
19.02
23.54
5.70
2.30
11.94

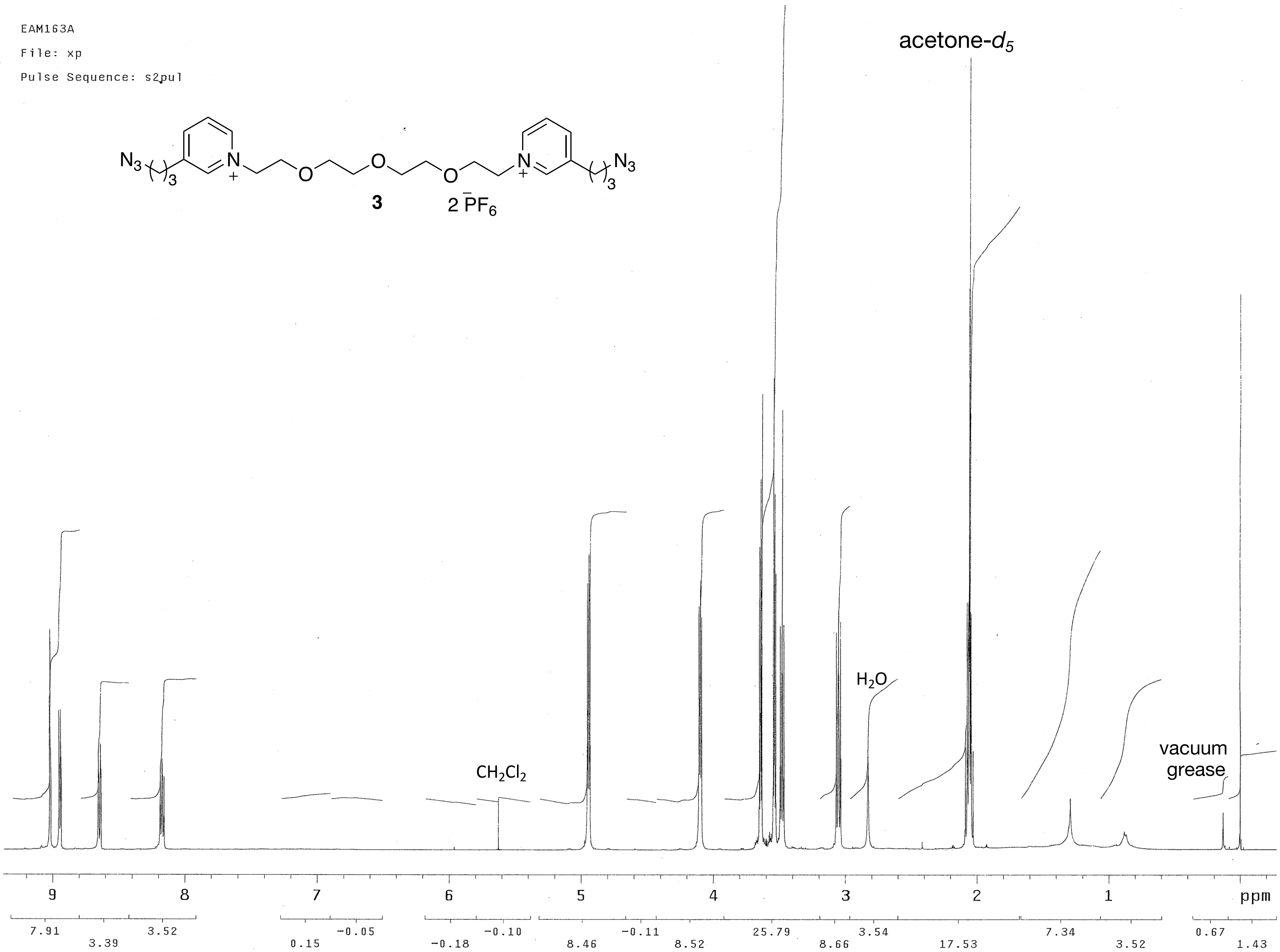
EAM163A
File: xp
Pulse Sequence: s2pul
3
2 PF6
acetone-d5
H2O
CH2Cl2
vacuum grease
9
8
7
6
5
4
3
2
1
ppm
7.91
3.39
3.52
0.15
-0.05
-0.18
-0.10
8.46
-0.11
8.52
25.79
8.66
3.54
17.53
7.34
3.52
0.67
1.43

Room temperature (~24 °C) spectrum

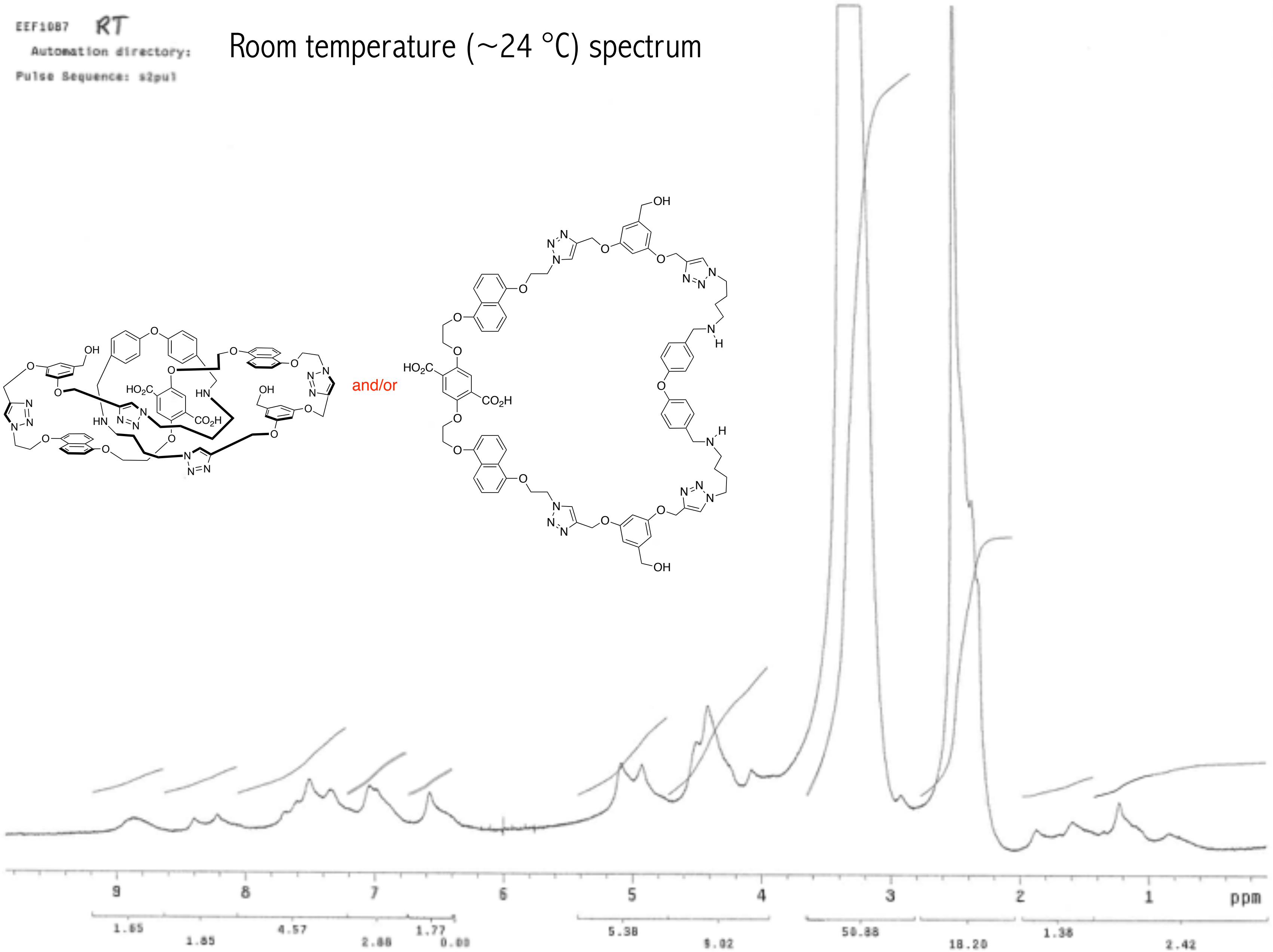

45 °C spectrum

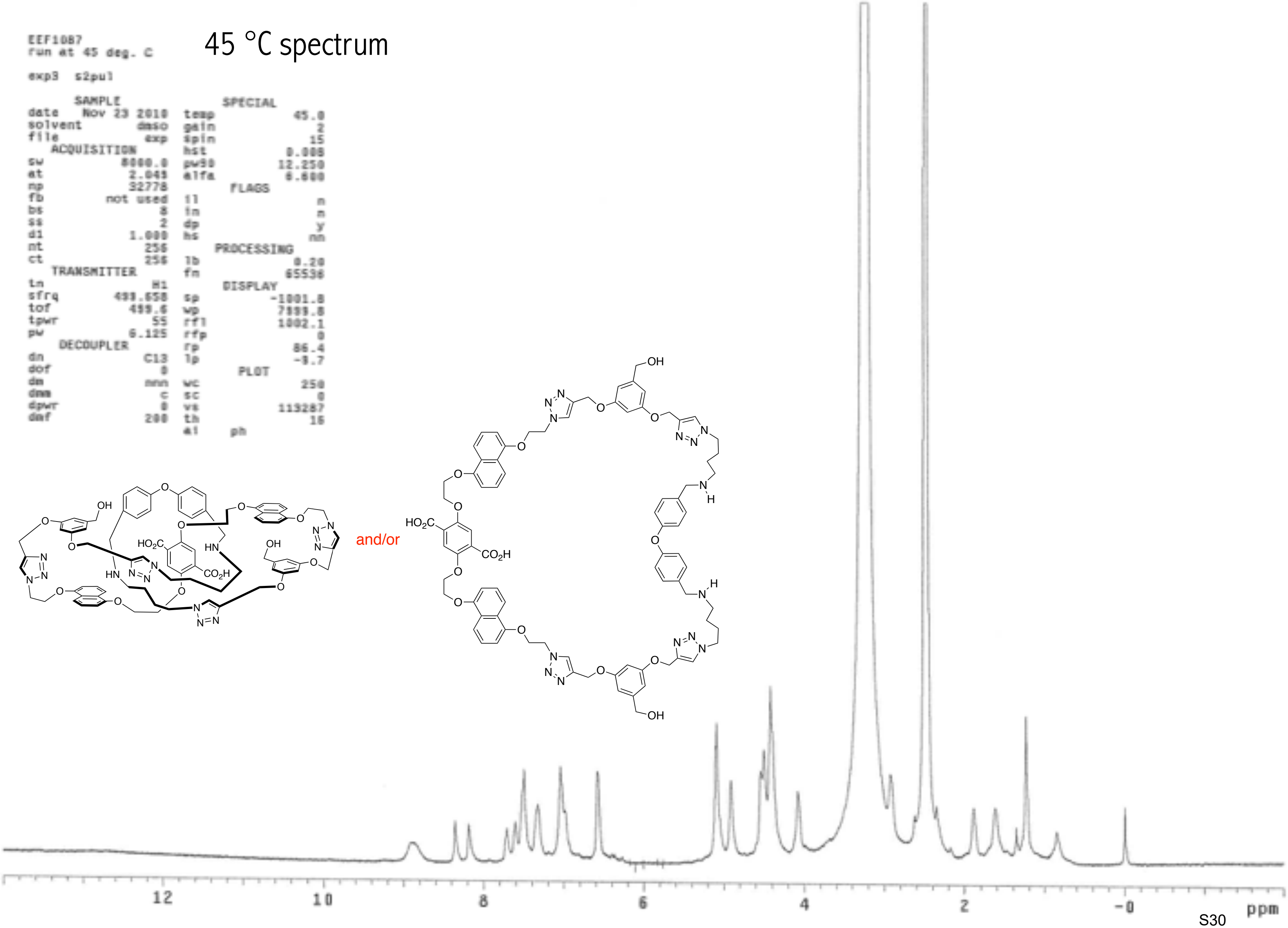

45 °C spectrum

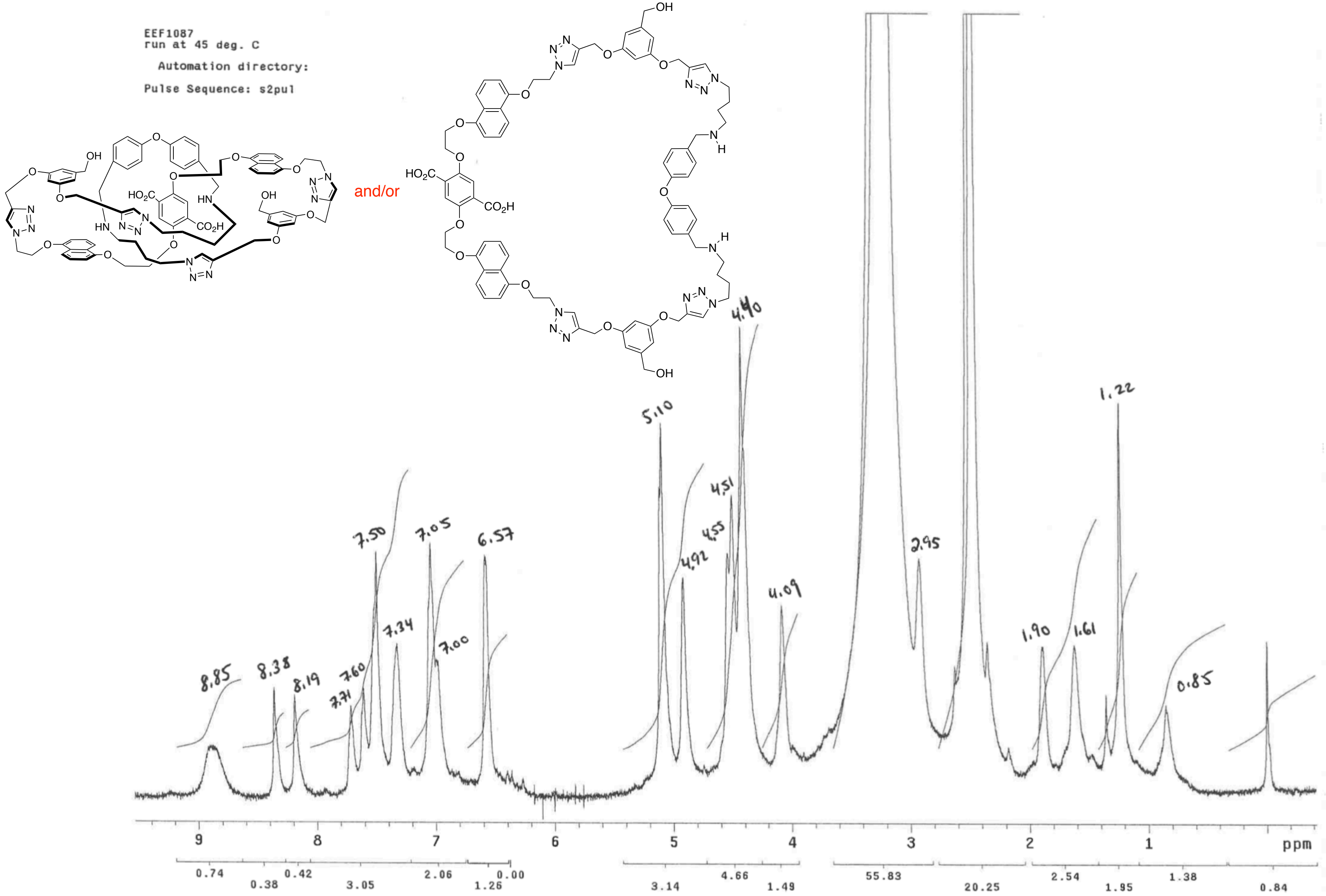

Product from the TLC sequence of **1** and **2**.

Applied Biosystems Voyager System 4289

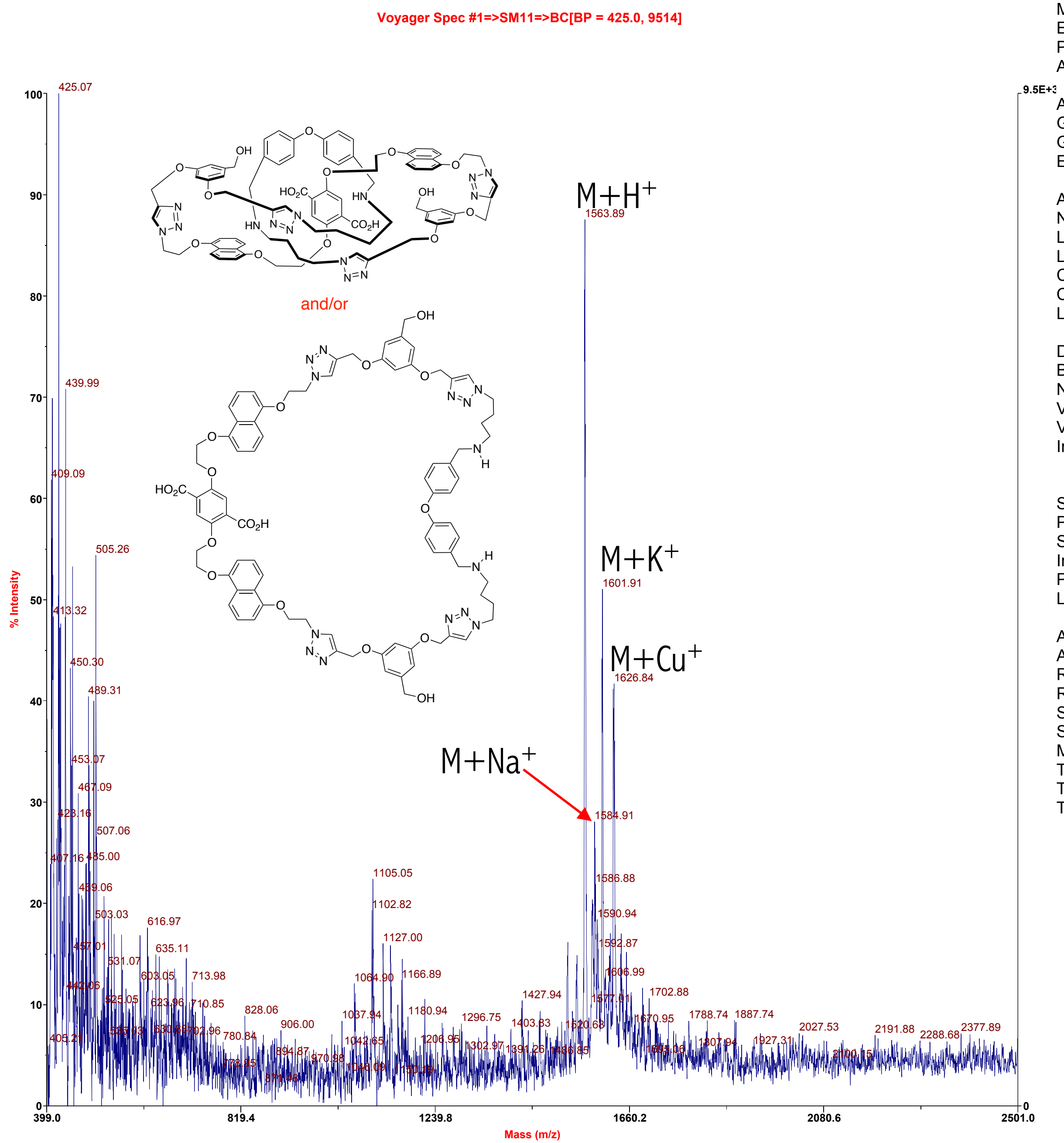

Mode of operation:	Linear
Extraction mode:	Delayed
Polarity:	Positive
Acquisition control:	Manual
Accelerating voltage:	20000 V
Grid voltage:	92.5%
Guide wire 0:	0.05%
Extraction delay time:	130 nsec
Acquisition mass range:	400 -- 2500 Da
Number of laser shots:	200/spectrum
Laser intensity:	1863
Laser Rep Rate:	20.0 Hz
Calibration type:	External -- H:\Mass_L\VIII\12_2010\C1206_AI_DHB.
Calibration matrix:	2,5-Dihydroxybenzoic acid
Low mass gate:	100 Da
Digitizer start time:	19.9155
Bin size:	0.5 nsec
Number of data points:	59375
Vertical scale:	500 mV
Vertical offset:	0%
Input bandwidth:	500 MHz
Sample well:	32
Plate ID:	100
Serial number:	4289
Instrument name:	Voyager-DE STR
Plate type filename:	C:\VOYAGER\100 well plate.plt
Lab name:	PE Biosystems
Absolute x-position:	4689.6
Absolute y-position:	32059.5
Relative x-position:	-1977.9
Relative y-posititon:	-8.01307
Shots in spectrum:	200
Source pressure:	2.206e-007
Mirror pressure:	5.581e-008
TC2 pressure:	0.01888
TIS gate width:	30
TIS flight length:	1167
